# A step-by-step guide to analyzing CAGE data using R/Bioconductor

**DOI:** 10.12688/f1000research.18456.1

**Published:** 2019-06-18

**Authors:** Malte Thodberg, Albin Sandelin

**Affiliations:** 1 Biotech Research and Innovation Centre, University of Copenhagen, Copenhagen, Denmark; 2 Section for Computational and RNA Biology, University of Copenhagen, Copenhagen, Denmark

**Keywords:** CAGE, TSS, Enhancer, Promoter, DE, Motifs

## Abstract

Cap Analysis of Gene Expression (CAGE) is one of the most popular 5'-end sequencing methods. In a single experiment, CAGE can be used to locate and quantify the expression of both Transcription Start Sites (TSSs) and enhancers. This is workflow is a case study on how to use the CAGEfightR package to orchestrate analysis of CAGE data within the Bioconductor project. This workflow starts from BigWig-files and covers both basic CAGE analyses such as identifying, quantifying and annotating TSSs and enhancers, advanced analysis such as finding interacting TSS-enhancer pairs and enhancer clusters, to differential expression analysis and alternative TSS usage. R-code, discussion and references are intertwined to help provide guidelines for future CAGE studies of the same kind.

## Background

Transcriptional regulation is one of the most important aspects of gene expression. Transcription Start Sites (TSSs) are focal points of this process: The TSS act as an integration point for a wide range of molecular cues from surrounding genomic areas to determine transcription and ultimately expression levels. These include proximal factors such as chromatin accessibility, chromatin modification, DNA methylation and transcription factor binding, and distal factors such as enhancer activity and chromatin confirmation ^1– 4^.

Cap Analysis of Gene Expression (CAGE) has emerged as one of the dominant high-throughput assays for studying TSSs ^5^. CAGE is based on “cap trapping”: capturing capped full-length RNAs and sequencing only the first 20–30 nucleotides from the 5’-end, so-called CAGE tags ^6^. When mapped to a reference genome, the 5’-ends of CAGE tag identify the actual TSS for respective RNA with basepair-level accuracy. Basepair-accurate TSSs identified this way are referred to as CAGE Transcription Start Sites (CTSSs). RNA polymerase rarely initiates from just a single nucleotide: this is manifested in CAGE data by the fact that CTSSs are mostly found in tightly spaced groups on the same strand. The majority of CAGE studies have merged such CTSSs into genomic blocks typically referred to as Tag Clusters (TCs), using a variety of clustering methods (see below). TCs are often interpreted as TSSs in the more general sense, given that most genes have many CTSSs, but only a few TCs that represent a few major transcripts with highly similar CTSSs ^7, 8^. Since the number of mapped CAGE tags in a given TC is indicative of the number of RNAs from that region, the number of CAGE tags falling in given TC can be seen as a measure of expression ^9^.

As CAGE tags can be mapped to a reference genome without the need for transcript annotations, it can detect TSSs of known mRNAs, but also mRNA from novel alternative TSSs (that might be condition or tissue dependent) ^7, 10^. Since CAGE captures all capped RNAs, it can also identify long non-coding RNA (lincRNA) ^11^ and enhancers RNA (eRNA). It was previously shown that active enhancers are characterized by balanced bidirectional transcription, making it possible to predict enhancer regions and quantify their expression levels from CAGE data alone ^12, 13^. Thus, CAGE data can predict the locations and activity of mRNAs, lincRNAs and enhancers in a single assay, providing a comprehensive view of transcriptional regulation across both inter- and intragenic regions.

Bioconductor contains a vast collection of tools for analyzing transcriptomics datasets, in particular the widely used RNA-Seq and microarray assays ^14^. Only a few packages are dedicated to analyzing 5’-end data in general and CAGE data in particular: *TSRchitect* ^15^, *icetea* ^16^, *CAGEr* ^17^ and *CAGEfightR* ^18^, see Table 1.

**Table 1.** Comparison of Bioconductor packages for CAGE data analysis.

Analysis	icetea	TSRchitect	CAGEr	CAGEfightR
Simplest input	FASTQ	BAM	BAM	BigWig
TSS calling	sliding window	X-means	distance or paraclu	slice-reduce
TSS shapes	-	+	+	+
Differential Expression	+	+	+	-
Enhancer calling	-	-	-	+
TSS-enhancer correlation	-	-	-	+
Super enhancers	-	-	-	+

CAGEr was the first package solely dedicated to the analysis of CAGE data and was recently updated to more closely adhere to Bioconductor S4-class standards. CAGEr takes as input aligned reads in the form of BAM-files and can identify, quantify, characterize and annotate TSSs. TSSs are found in individual samples using either simple clustering of CTSSs (greedy or distance-based clustering) or the more advanced density-based paraclu clustering method ^19^, and can be aggregated across samples to a set of consensus clusters. Several specialized routines for CAGE data is available, such as power law normalization of CTSS counts and fine-grained TSS shifts. Finally, CAGEr offers easy interface to the large collection of CAGE data from the FANTOM consortium ^10^. TSRchitect and icetea are two more recent additions to Bioconductor. While being less comprehensive, they aim to be more general and handle more types of 5’-end methods that are conceptually similar to CAGE (RAMPAGE, PEAT, PRO-Cap, etc. ^5^). Both packages can identify, quantify and annotate TSSs, with TSRchitect using an X-means algorithm and icetea using a sliding window approach. icetea offers the unique feature of mapping reads to a reference genome by interfacing with *Rsubread*. Both CAGEr, TSRchictet and icetea offers built-in capabilities for differential expression (DE) analysis via the popular *DESeq2* or *edgeR* packages ^20, 21^.

CAGEfightR is a recent addition to Bioconductor focused on analyzing CAGE data, but applicable to most 5’-end data. It aims to be general and flexible to allow for easy interfacing with the wealth of other Bioconductor packages. CAGEfightR takes CTSSs stored in BigWig-files as input and uses only standard Bioconductor S4-classes ( *GenomicRanges*, *SummarizedExperiment*, *InteractionSet* ^22, 23^) making it easy for users to learn and combine CAGEfightR with functions from other Bioconductor packages (e.g. instead of providing custom wrappers around other packages such as differential expression analysis). In addition to TSS analysis, CAGEfightR is the only package on Bioconductor to also offer functions for enhancer analysis based on CAGE (and similarly scoped) data. This includes enhancer identification and quantification, linking enhancers to TSSs via correlation of expression and finding enhancer clusters, often referred to as stretch- or super enhancers.

In this workflow, we illustrate how the CAGEfightR package can be used to orchestrate an end-to-end analysis of CAGE data by making it easy to interface with a wide range of different Bioconductor packages. Highlights include TSS and enhancer candidate identification, differential expression, alternative TSS usage, enrichment of motifs, GO/KEGG terms and calculating TSS-enhancer correlations.

## Methods

### Dataset

This workflow uses data from *“Identification of Gene Transcription Start Sites and Enhancers Responding to Pulmonary Carbon Nanotube Exposure in Vivo”* by Bornholdt *et al* ^24^. This study uses mouse as a model system to investigate how nanotubes affect lung tissue when inhaled. Inhaled nanotubes were previously found to produce a similar response to asbestos, potentially triggering an inflammatory response in the lung tissue leading to drastic changes in gene expression.

The dataset consists of CAGE data from mouse lung biopsies: 5 mice whose lungs were instilled with water (Ctrl) and 6 mice wholes lungs were instilled with nanotubes (Nano), with CTSSs for each sample stored in BigWig-files, shown in Table 2:

**Table 2.** Overview of samples in the nanotube exposure experiment.

Group	Biological Replicates
Ctrl	5 mice
Nano	6 mice

The data is acquired via the nanotubes data package:


library(nanotubes)


### R-packages

This workflow uses a large number of R-packages: Bioconductor packages are primarily used for data analysis while packages from the tidyverse are used to wrangle and plot the results. All these packages are loaded prior to beginning the workflow:


# CRAN packages for data manipulation and plotting 
library(knitr)                                     
library(kableExtra)                                
library(pheatmap)                                  
library(ggseqlogo)                                 
library(viridis)                                   
library(magrittr)                                  
library(ggforce)                                   
library(ggthemes)                                  
library(tidyverse)                                 

# CAGEfightR and related packages                  
library(CAGEfightR)                                
library(GenomicRanges)                             
library(SummarizedExperiment)                      
library(GenomicFeatures)                           
library(BiocParallel)                              
library(InteractionSet)                            
library(Gviz)                                      

# Bioconductor packages for differential expression
library(DESeq2)                                    
library(limma)                                     
library(edgeR)                                     
library(sva)                                       

# Bioconductor packages for enrichment analyses    
library(TFBSTools)                                 
library(motifmatchr)                               
library(pathview)                                  

# Bioconductor data packages                       
library(BSgenome.Mmusculus.UCSC.mm9)               
library(TxDb.Mmusculus.UCSC.mm9.knownGene)         
library(org.Mm.eg.db)                              
library(JASPAR2016)                                


We also set some script-wide settings for later convenience:


# Rename these for easier access                              
bsg <- BSgenome.Mmusculus.UCSC.mm9                            
txdb <- TxDb.Mmusculus.UCSC.mm9.knownGene                     
odb <- org.Mm.eg.db                                           

# Script wide settings                                        
register(MulticoreParam(3)) # Parallel execution when possible
theme_set(theme_light()) # White theme for ggplot2 figures    


### Workflow

The workflow is divided into 3 parts covering different parts of a typical CAGE data analysis:

1. Shows how to use CAGEfightR to import CTSSs and find and quantify TSS and enhancer candidates.
2. Shows examples of how to perform genomic analyses of CAGE dusters using core Bioconductor packages such as *GenomicRanges* and *Biostrings*. This part covers typical analyses made from CAGE data, from summarizing cluster annotation, TSS shapes and core promoter sequence analysis to more advanced spatial analyses (finding TSS-enhancer correlation links and clusters of enhancers).
3. Shows how CAGEfightR can be used to prepare data for differential expression analysis with popular R packages, including *DESeq2*, *limma* and *edgeR* ^20, 21, 25^. Borrowing from RNA-Seq terminology, differential expression can be assessed at multiple different levels: Tag cluster- and enhancer-level, gene-level and differential TSS usage ^26^. Once differential expression results have been obtained, they can be combined with other sources of information such as motifs from JASPAR ^27^ and GO/KEGG terms ^28, 29, 30^.

### Part 1: Locating, quantifying and annotating TSSs and enhancers

CAGEfightR starts analysis from CTSSs, which is the number of CAGE tag 5’-ends mapping to each basepair (bp) in the genome. CTSSs are normally stored as either BED-files or BigWig-files. CAGEfightR works on BigWig-files, since these can be efficiently imported and allow for random access.

Before starting the analysis, we recommend gathering all information (Filenames, groups, batches, preparation data, etc.) about the samples to be analyzed in a single data.frame, sometimes called the *design matrix*. CAGEfightR can keep track of the design matrix throughout the analysis:


data(nanotubes)                                                              
kable(nanotubes,                                                             
      caption = "The initial design matrix for the nanotubes experiment") %>%
    kable_styling(latex_options = "hold_position")                           


**Table 3.** The initial design matrix for the nanotubes experiment.

	Class	Name	BigWigPlus	BigWigMinus
C547	Ctrl	C547	mm9.CAGE_7J7P_NANO_KON_547.plus. bw	mm9.CAGE_7J7P_NANO_KON_547.minus. bw
C548	Ctrl	C548	mm9.CAGE_ULAC_NANO_KON_548. plus.bw	mm9.CAGE_ULAC_NANO_KON_548.minus. bw
C549	Ctrl	C549	mm9.CAGE_YM4F_Nano_KON_549.plus. bw	mm9.CAGE_YM4F_Nano_KON_549.minus. bw
C559	Ctrl	C559	mm9.CAGE_RSAM_NANO_559.plus.bw	mm9.CAGE_RSAM_NANO_559.minus.bw
C560	Ctrl	C560	mm9.CAGE_CCLF_NANO_560.plus.bw	mm9.CAGE_CCLF_NANO_560.minus.bw
N13	Nano	N13	mm9.CAGE_KTRA_Nano_13.plus.bw	mm9.CAGE_KTRA_Nano_13.minus.bw
N14	Nano	N14	mm9.CAGE_RSAM_NANO_14.plus.bw	mm9.CAGE_RSAM_NANO_14.minus.bw
N15	Nano	N15	mm9.CAGE_RFQS_Nano_15.plus.bw	mm9.CAGE_RFQS_Nano_15.minus.bw
N16	Nano	N16	mm9.CAGE_CCLF_NANO_16.plus.bw	mm9.CAGE_CCLF_NANO_16.minus.bw
N17	Nano	N17	mm9.CAGE_RSAM_NANO_17.plus.bw	mm9.CAGE_RSAM_NANO_17.minus.bw
N18	Nano	N18	mm9.CAGE_CCLF_NANO_18.plus.bw	mm9.CAGE_CCLF_NANO_18.minus.bw

***Importing CTSSs. *** We need to tell CAGEfightR where to find the BigWig-files containing CTSSs on the hard drive. Normally, one would supply the paths to each file (e.g. / CAGEdata/BigWigFiles/Sample1_plus.bw), but here we will use data from the nanotubes data package:


# Setup paths to file on hard drive                      
bw_plus <- system.file("extdata", nanotubes$BigWigPlus,  
                        package = "nanotubes",           
                        mustWork = TRUE)                 
bw_minus <- system.file("extdata", nanotubes$BigWigMinus,
                        package = "nanotubes",           
                        mustWork = TRUE)                 

# Save as named BigWigFileList                           
bw_plus <- BigWigFileList(bw_plus)                       
bw_minus <- BigWigFileList(bw_minus)                     
names(bw_plus) <- names(bw_minus) <- nanotubes$Name      


The first step is quantifying CTSS usage across all samples. This is often one of the most time consuming step in a CAGEfightR analysis, but it can be speed up by using multiple cores (if available, see Materials and Methods). We also need to specify the genome, which we can get from the *BSgenome.Mmusculus.UCSC.mm9* genome package:


CTSSs <- quantifyCTSSs(plusStrand = bw_plus,                         
                       minusStrand = bw_minus,                       
                       genome = seqinfo(bsg),                        
                       design = nanotubes)                           
#> Checking supplied genome compatibility...                         
#> Iterating over 28 genomic tiles in 11 samples using 3 worker(s)...
#> Importing CTSSs from plus strand...                               
#> Registered S3 method overwritten by ’pryr’:                       
#>   method      from                                                
#>   print.bytes Rcpp                                                
#> Importing CTSSs from minus strand...                              
#> Merging strands...                                                
#> ### CTSS summary ###                                              
#> Number of samples: 11                                             
#> Number of CTSSs: 9.339 millions                                   
#> Sparsity: 81.68 %                                                 
#> Final object size: 282 MB                                         


The circa 9 million CTSSs are stored as RangedSummarizedExperiment, which is the standard representation of high-throughput experiments in Bioconductor. We can inspect both the ranges and the CTSS counts:


# Get a summary                                                   
CTSSs                                                             
#> class: RangedSummarizedExperiment                              
#> dim: 9338802 11                                                
#> metadata(0):                                                   
#> assays(1): counts                                              
#> rownames: NULL                                                 
#> rowData names(0):                                              
#> colnames(11): C547 C548 ... N17 N18                            
#> colData names(4): Class Name BigWigPlus BigWigMinus            

# Extract CTSS positions                                          
rowRanges(CTSSs)                                                  
#> GPos object with 9338802 positions and 0 metadata columns:     
#>                 seqnames       pos strand                      
#>                    <Rle> <integer>  <Rle>                      
#>         [1]         chr1   3024556      +                      
#>         [2]         chr1   3025704      +                      
#>         [3]         chr1   3025705      +                      
#>         [4]         chr1   3028283      +                      
#>         [5]         chr1   3146133      +                      
#>         ...          ...       ...    ...                      
#>   [9338798] chrUn_random   5810899      -                      
#>   [9338799] chrUn_random   5813784      -                      
#>   [9338800] chrUn_random   5880838      -                      
#>   [9338801] chrUn_random   5893536      -                      
#>   [9338802] chrUn_random   5894263      -                      
#>   -------                                                      
#>   seqinfo: 35 sequences (1 circular) from mm9 genome           

# Extract CTSS counts                                             
assay(CTSSs, "counts") %>%                                        
    head                                                          
#> 6 x 11 sparse Matrix of class "dgCMatrix"                      
#>    [[ suppressing 11 column names ’C547’, ’C548’, ’C549’ ... ]]
#>                                                                
#> [1,] . . 1 . . . . . . . .                                     
#> [2,] . . . 1 . . . . . . .                                     
#> [3,] . . . . 1 . . . . . .                                     
#> [4,] . . . . 1 . . . . . .                                     
#> [5,] . . . . . . 1 . . . .                                     
#> [6,] . 1 . . . . . . . . .                                     


***Unidirectional and bidirectional clustering for finding TSS and enhancer candidates.*** CAGEfightR finds clusters by calculating the pooled CTSS signal across all samples: We first normalize CTSSs count in each sample to Tags-Per-Million (TPM) values, and them sum TPM values across samples:


CTSSs <- CTSSs %>%             
    calcTPM() %>%              
    calcPooled()               
#> Calculating library sizes...
#> Calculating TPM...          


This will add several new pieces of information to CTSSs: The total number of tags in each library, a new assay called TPM, and the pooled signal for each CTSS.

We can use *unidirectional clustering* to locate unidirectional clusters, often simply called Tag Clusters (TCs), which are candidates for TSSs. The quickTSSs will both locate and quantify TCs in a single function call:


TCs <- quickTSSs(CTSSs)               
#> Using existing score column!       
#>                                    
#>  - Running clusterUnidirectionally:
#> Splitting by strand...             
#> Slice-reduce to find clusters...   
#> Calculating statistics...          
#> Preparing output...                
#> Tag clustering summary:            
#>   Width   Count Percent            
#>   Total 3602099 1e+02 %            
#>     >=1 2983433 8e+01 %            
#>    >=10  577786 2e+01 %            
#>   >=100   40842 1e+00 %            
#>  >=1000      38 1e-03 %            
#>                                    
#>  - Running quantifyClusters:       
#> Finding overlaps...                
#> Aggregating within clusters...     


**Note:** quickTSSs runs CAGEfightR with default settings. If you have larger or more noisy datasets you most likely want to do a more robust analysis with different settings. See the CAGEfightR vignette for more information.

Many of the identified TCs will only be very lowly expressed. To obtain likely biologically relevant TSSs, we keep only TSSs expressed at more than 1 TPM in at least 5 samples (5 samples being the size of the smallest experimental group):


TSSs <- TCs %>%                                                             
    calcTPM() %>%                                                           
    subsetBySupport(inputAssay="TPM",                                       
                    unexpressed=1,                                          
                    minSamples=4)                                           
#> Calculating library sizes...                                             
#> Warning in calcTotalTags(object = object, inputAssay = inputAssay,       
#> outputColumn = outputColumn): object already has a column named totalTags
#> in colData: It will be overwritten!                                      
#> Calculating TPM...                                                       
#> Calculating support...                                                   
#> Subsetting...                                                            
#> Removed 3573214 out of 3602099 regions (99.2%)                           


This removed a large number of very lowly expressed TCs, leaving us with almost 30.000 TSSs candidates for analysis.

Then we turn to *bidirectional clustering* for identifying bidirectional clusters (BCs), which are candidate for enhancers. Similarly, we can use quickEnhancers to locate and quantify BCs:


BCs <- quickEnhancers(CTSSs)                       
#> Using existing score column!                    
#>                                                 
#>  - Running clusterBidirectionally:              
#> Pre-filtering bidirectional candidate regions...
#> Retaining for analysis: 68.3%                   
#> Splitting by strand...                          
#> Calculating windowed coverage on plus strand... 
#> Calculating windowed coverage on minus strand...
#> Calculating balance score...                    
#> Slice-reduce to find bidirectional clusters...  
#> Calculating statistics...                       
#> Preparing output...                             
#> # Bidirectional clustering summary:             
#> Number of bidirectional clusters: 106779        
#> Maximum balance score: 1                        
#> Minimum balance score: 0.950001090872574        
#> Maximum width: 1866                             
#> Minimum width: 401                              
#>                                                 
#>  - Running subsetByBidirectionality:            
#> Calculating bidirectionality...                 
#> Subsetting...                                   
#> Removed 73250 out of 106779 regions (68.6%)     
#>                                                 
#>  - Running quantifyClusters:                    
#> Finding overlaps...                             
#> Aggregating within clusters...                  


**Note:** quickEnhancers runs CAGEfightR with default settings. If you have larger or more noisy datasets you most likely want to do a more robust analysis with different settings. See the CAGEfightR vignette for more information.

Again, we are not usually interested in very lowly expressed BCs. As they are overall lowly expressed, we will simply filter out BCs without at least 1 count in 5 samples:


BCs <- subsetBySupport(BCs, inputAssay="counts", unexpressed=0, minSamples=4)
#> Calculating support...                                                    
#> Subsetting...                                                             
#> Removed 20017 out of 33529 regions (59.7%)                                


***Annotating clusters with transcript models. ***After having located unidirectional and bidirectional clusters, we can annotate them according to known transcript and gene models. These types of annotation are store via TxDb-objects in Bioconductor. Here we will simply use UCSC transcripts included in the *TxDb.Mmusculus.UCSC.mm9.knownGene* package, but the CAGEfightR vignette includes examples of how to obtain a TxDb object from other sources (GFF/GTF files, AnnotationHub, etc.).

Starting with the TSS candidates, we can not only annotate a TSS with overlapping transcripts, but also in what *part* of a transcript a TSS lies by using a hierarchical annotation scheme. As some TSSs might be very wide, we only use the TSS peak for annotation purposes:


# Annotate with transcript IDs                           
TSSs <- assignTxID(TSSs, txModels = txdb,swap="thick")  
#> Extracting transcripts...                             
#> Finding hierachical overlaps...                       
#> ### Overlap Summary: ###                              
#> Features overlapping transcripts: 87.65 %             
#> Number of unique transcripts: 31898                   

# Annotate with transcript context                       
TSSs <- assignTxType(TSSs, txModels = txdb, swap="thick")
#> Finding hierachical overlaps with swapped ranges...   
#> ### Overlap summary: ###                              
#>       txType count percentage                         
#> 1   promoter 13395       46.4                         
#> 2   proximal  2246        7.8                         
#> 3    fiveUTR  2112        7.3                         
#> 4   threeUTR  1200        4.2                         
#> 5        CDS  3356       11.6                         
#> 6       exon   161        0.6                         
#> 7     intron  2810        9.7                         
#> 8  antisense  1294        4.5                         
#> 9 intergenic  2311        8.0                         


Almost half of TSSs were found at annotated promoters, while the other half is novel compared to the UCSC known transcripts.

Transcript annotation is particularly useful for enhancer candidates, as bidirectional clustering might also detect bidirectional promoters. Therefore, a commonly used filtering approached is to only consider BCs in intergenic or intronic regions as enhancer candidates:


# Annotate with transcript context                             
BCs <- assignTxType(BCs, txModels = txdb, swap="thick")        
#> Finding hierachical overlaps with swapped ranges...         
#> ### Overlap summary: ###                                    
#>       txType count percentage                               
#> 1   promoter   766        5.7                               
#> 2   proximal  1649       12.2                               
#> 3    fiveUTR    67        0.5                               
#> 4   threeUTR   596        4.4                               
#> 5        CDS   420        3.1                               
#> 6       exon    71        0.5                               
#> 7     intron  6815       50.4                               
#> 8  antisense     0        0.0                               
#> 9 intergenic  3128       23.1                               

                            
# Keep only non-exonic BCs as enhancer candidates              
Enhancers <- subset(BCs, txType %in% c("intergenic", "intron"))


This leaves almost 10000 enhancer candidates for analysis.

***Merging into a single dataset. ***For many downstream analyses, in particular normalization and differential expression, it is useful to combine both TSS and enhancers candidates into a single dataset. This ensures that TSSs and enhancers do not overlap, so each CAGE tag is only counted once.

We must first ensure that the enhancer and TSS candidates have the same information attached to them, since CAGEfightR will only allow merging of clusters if they have the same sample and cluster information:


# Clean colData                             
TSSs$totalTags <- NULL                      
Enhancers$totalTags <- NULL                 

# Clean rowData                             
rowData(TSSs)$balance <- NA                 
rowData(TSSs)$bidirectionality <- NA        
rowData(Enhancers)$txID <- NA               

# Add labels for making later retrieval easy
rowData(TSSs)$clusterType <- "TSS"          
rowData(Enhancers)$clusterType <- "Enhancer"


Then the clusters can be merged: As enhancers are the most complicated type, we keep only enhancers if a TSS and enhancer overlaps:


RSE <- combineClusters(object1=TSSs,                                                           
                       object2 = Enhancers,                                                    
                       removeIfOverlapping="object1")                                          
#> Removing overlapping features from object1: 374                                             
#> Keeping assays: counts                                                                      
#> Keeping columns: score, thick, support, txID, txType, balance, bidirectionality, clusterType
#> Merging metadata...                                                                         
#> Stacking and re-sorting...                                                                  


We finally calculate the total number of tags and TPM-scaled counts for the final merged dataset:


RSE <- calcTPM(RSE)            
#> Calculating library sizes...
#> Calculating TPM...          


### Part 2: Genomic analysis of TSSs and enhancers

***Genome-browser figures of TSSs and enhancers. ***First we can simply plot some examples of TSSs and enhancers in a genome browser style figure using the Gviz package ^31^. It takes a bit of code to setup, but the resulting tracks can be reused for later examples:


# Genome track                                   
axis_track <- GenomeAxisTrack()                  

# Annotation track                               
tx_track <- GeneRegionTrack(txdb,                
                            name = "Gene Models",
                            col = NA,            
                            fill = "bisque4",    
                            shape = "arrow",     
                            showId = TRUE)       


A good general strategy for quickly generating genome browser plots is to first define a region of interest, and then only plotting data within that region using subsetByOverlaps. The following code demonstrates this using the first TSS:


# Extract 100 bp around the first TSS.        
plot_region <- RSE %>%                        
    rowRanges %>%                             
    subset(clusterType == "TSS") %>%          
    .[1] %>%                                  
    add(100) %>%                              
    unstrand()                                

# CTSSs track                                 
ctss_track <- CTSSs %>%                       
    rowRanges %>%                             
    subsetByOverlaps(plot_region) %>%         
    trackCTSS(name = "CTSSs")                 
#> Splitting pooled signal by strand...       
#> Preparing track...                         

# Cluster track                               
cluster_track <- RSE %>%                      
    subsetByOverlaps(plot_region) %>%         
    trackClusters(name = "Clusters",          
                  col = NA,                   
                  showId=TRUE)                
#> Setting thick and thin features...         
#> Merging and sorting...                     
#> Preparing track...                         

# Plot at tracks together                     
plotTracks(list(axis_track,                   
                ctss_track,                   
                cluster_track,                
                tx_track),                    
           from = start(plot_region),         
           to=end(plot_region),               
           chromosome = seqnames(plot_region))


**Figure 1.**
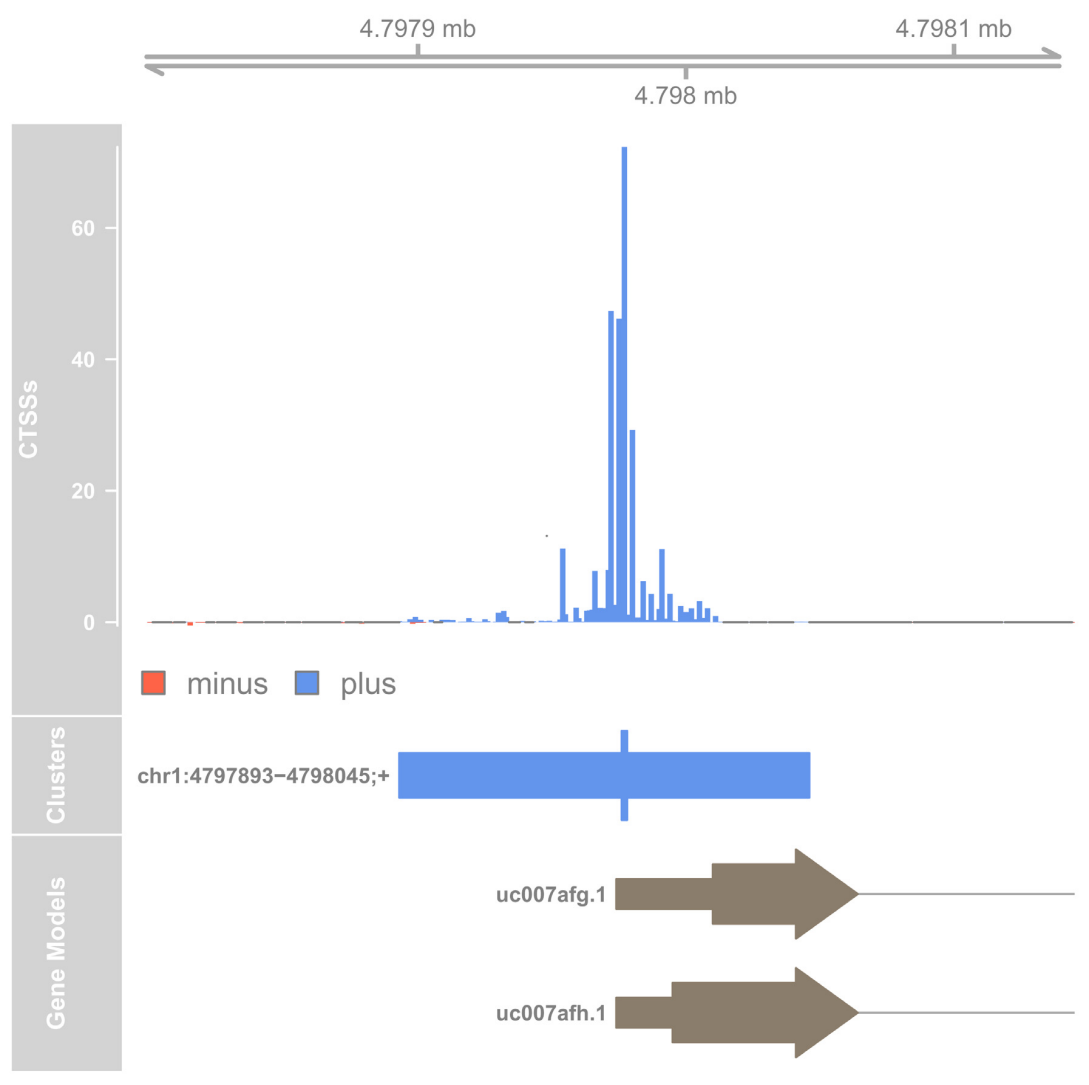
Genome browser example of TSS candidate.

The top track shows the pooled CTSS signal and the middle track shows the identified TC with the thick bar indicating the TSS peak (the overall most used CTSSs within the TC). The bottom track shows the known transcript model at this genomic location. In this case, the CAGE-defined TSS corresponds well to the annotation.

We can also plot the first enhancer:


# Make plotting region                                      
plot_region <- RSE %>%                                      
    rowRanges %>%                                           
    subset(clusterType == "Enhancer") %>%                   
    .[1] %>%                                                
    add(100) %>%                                            
    unstrand()                                              

# CTSSs track                                               
ctss_track <- CTSSs %>%                                     
    rowRanges %>%                                           
    subsetByOverlaps(plot_region) %>%                       
    trackCTSS(name = "CTSSs")                               
#> Splitting pooled signal by strand...                     
#> Preparing track...                                       

# Cluster track                                             
cluster_track <- RSE %>%                                    
    rowRanges %>%                                           
    subsetByOverlaps(plot_region) %>%                       
    trackClusters(name = "Clusters",                        
                  col = NA,                                 
                  showId=TRUE)                              
#> Setting thick and thin features...                       
#> Merging and sorting...                                   
#> Preparing track...                                       

# Plot at tracks together                                   
plotTracks(list(axis_track,                                 
                ctss_track,                                 
                cluster_track,                              
                tx_track),                                  
           from = start(plot_region),                       
           to=end(plot_region),                             
           chromosome = as.character(seqnames(plot_region)))


**Figure 2.**
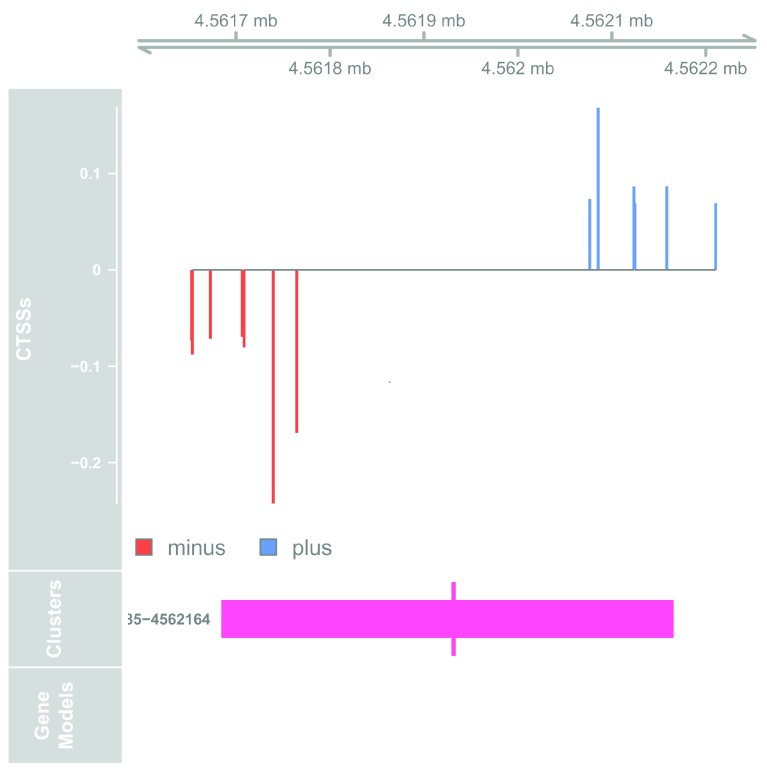
Genome browser example of enhancer candidate.

Here we see the bidirectional pattern characteristic of active enhancers. The bidirectional cluster is seen in the middle track, with the midpoint in thick marking the maximally balanced point within the bidirectional cluster.

***Location and expression of TSSs and enhancers. ***In addition to looking at single examples of TSSs and enhancers, we also want to get an overview of the number and expression of clusters in relation to transcript annotation. First we extract all of the necessary data from the RangedSummarizedExperiment into an ordinary data.frame:


cluster_info <- RSE %>%
    rowData() %>%      
    as.data.frame()    


Then we use *ggplot2* to plot the number and expression levels of clusters in each annotation category:


# Number of clusters                                        
ggplot(cluster_info, aes(x=txType, fill=clusterType)) +     
    geom_bar(alpha=0.75, position="dodge", color="black") + 
    scale_fill_colorblind("Cluster type") +                 
    labs(x="Cluster annotation", y="Frequency") +           
    theme(axis.text.x = element_text(angle = 90, hjust = 1))


**Figure 3.**
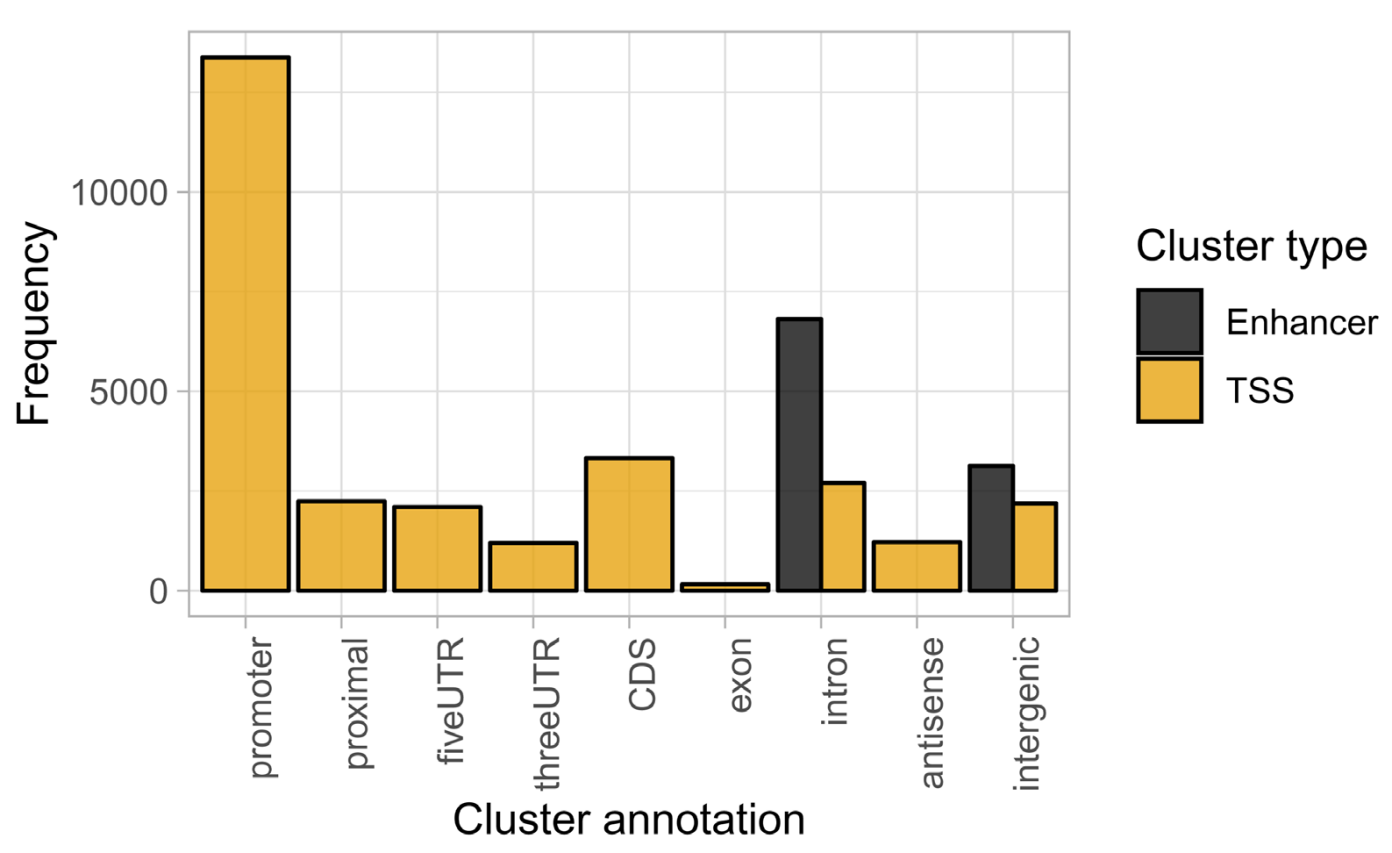
Number of clusters within each annotation category.


# Expression of clusters                                                 
ggplot(cluster_info, aes(x=txType,                                       
                         y=log2(score/ncol(RSE)),                        
                         fill=clusterType)) +                            
    geom_violin(alpha=0.75, draw_quantiles = c(0.25, 0.50, 0.75)) +      
    scale_fill_colorblind("Cluster type") +                              
    labs(x="Cluster annotation", y="log2(TPM)") +                        
    theme(axis.text.x = element_text(angle = 90, hjust = 1))             
#> Warning in regularize.values(x, y, ties, missing(ties)): collapsing to
#> unique ’x’ values                                                     


**Figure 4.**
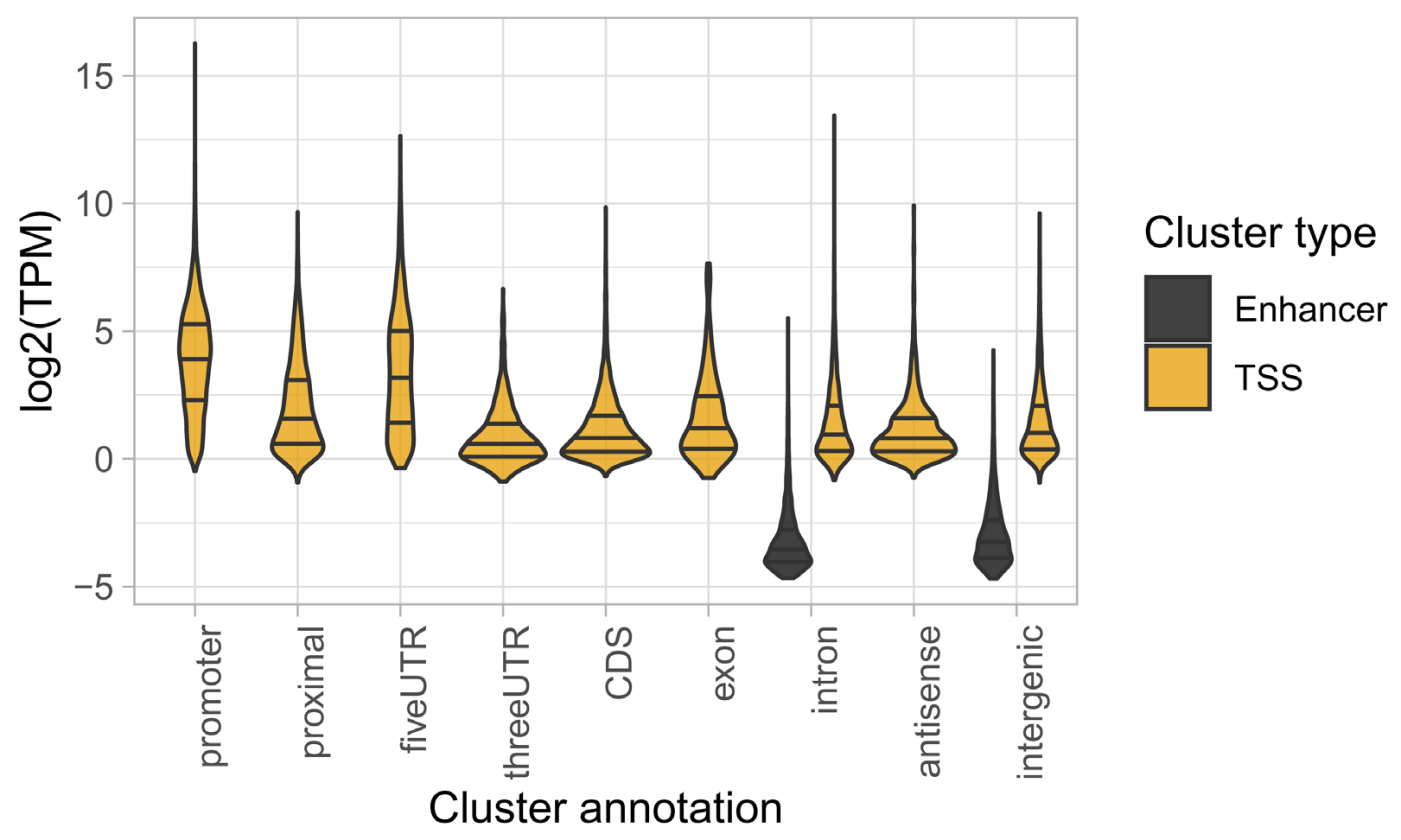
Expression of clusters within each annotation category.

We find that TSSs at annotated promoters are generally highly expressed. Most novel TSSs are expresse d at lower levels, except for some TSSs in 5’-UTRs. Enhancers are expressed at much lower levels than TSSs.

***Analysing TSS shapes and sequences. ***A classic analysis of CAGE data is to divide TSSs into *Sharp* and *Broad* classes, which show different core promoter regions and different expression patterns across tissues ^7^.

CAGEfightR can calculate several *shape statistics* that summarizes the shape of a TSS. The Interquartile Range (IQR) can be used to find sharp and broad TSSs. As lowly expressed TSSs cannot show much variation in shape due to their low width and number of tags, we here focused on highly expressed TSSs (average TPM >= 10):


# Select highly expressed TSSs                                         
highTSSs <- subset(RSE, clusterType == ’TSS’ & score / ncol(RSE) >= 10)

# Calculate IQR as 10%-90% interval                                    
highTSSs <- calcShape(highTSSs,                                        
                      pooled=CTSSs,                                    
                      shapeFunction=shapeIQR,                          
                      lower = 0.10,                                    
                      upper = 0.90)                                    
#> Splitting by strand...                                              
#> Applying function to each cluster...                                
#> Preparing output output...                                          


We can then plot the bimodal distribution of IQRs. We use a zoom-in panel to highlight the distinction between the two classes:


highTSSs %>%                                                                   
    rowData %>%                                                                
    as.data.frame %>%                                                          
    ggplot(aes(x=IQR)) +                                                       
    geom_histogram(binwidth=1, fill="hotpink", alpha=0.75) +                   
    geom_vline(xintercept = 10, linetype="dashed", alpha=0.75, color="black") +
    facet_zoom(xlim = c(0,100)) +                                              
    labs(x="10-90% IQR", y="Frequency")                                        


**Figure 5.**
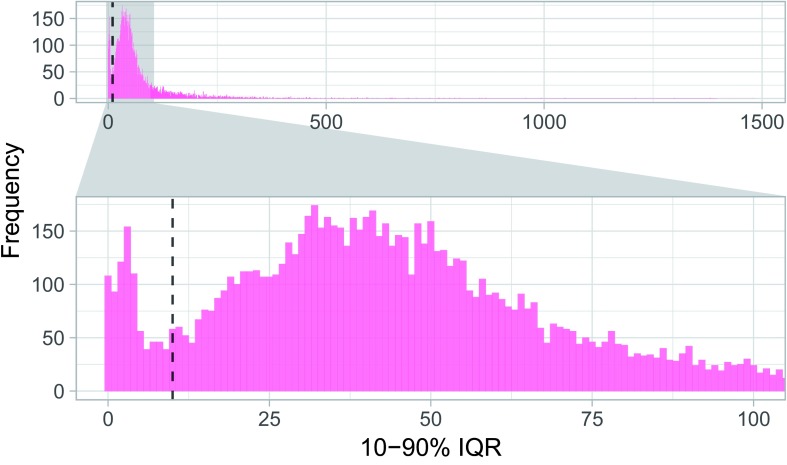
Bimodal distribution of Interquartile Ranges (IQRs) of highly expressed TSSs.

We see most TSSs are either below or above 10 bp IQR (dashed line), so we use this cutoff to classify TSSs into Sharp and Broad:


# Divide into groups                                                           
rowData(highTSSs)$shape <- ifelse(rowData(highTSSs)$IQR < 10, "Sharp", "Broad")

# Count group sizes                                                            
table(rowData(highTSSs)$shape)                                                 
#>                                                                             
#> Broad Sharp                                                                 
#>  9555   812                                                                 


We can now investigate the core promoters sequences of the two classes of TSSs. We first need to extract the sequences for each TSS: We define this as the TSS peak -40/+10 bp and extract them from using the *BSgenome.Mmusculus.UCSC.mm10* genome package:


promoter_seqs <- highTSSs %>%                
    rowRanges() %>%                          
    swapRanges() %>%                         
    promoters(upstream=40, downstream=10) %>%
    getSeq(bsg, .)                           


This returns a DNAStringSet-object which we can plot as a sequence logo ^32^ via the *ggseqlogo* package ^33^:


promoter_seqs %>%                      
    as.character %>%                   
    split(rowData(highTSSs)$shape) %>% 
    ggseqlogo(data=., ncol=2, nrow=1) +
    theme_logo() +                     
    theme(axis.title.x=element_blank(),
          axis.text.x=element_blank(), 
          axis.ticks.x=element_blank())


**Figure 6.**
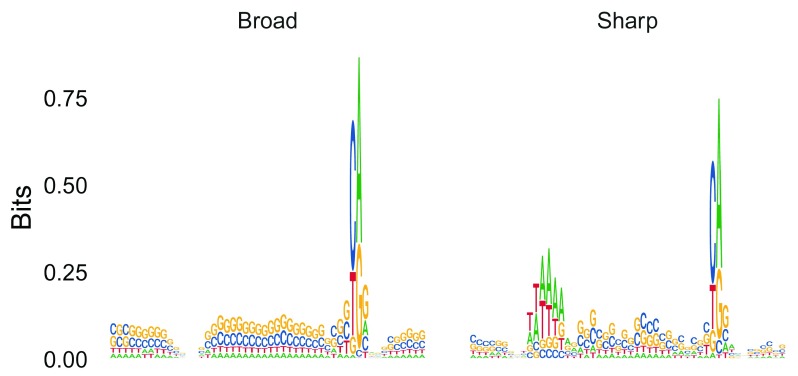
Sequence logos of core promoter regions of Sharp and Broad classes of TSSs.

As expected, we observe that Sharp TSSs tend to have a stronger TATA-box upstream of the TSS compared to Broad TSSs.

***Finding candidates for interacting TSSs and enhancers. ***In addition to simply identifying enhancers, it is also interesting to try infer what genes they might be regulating. CAGE data can itself not provide direct evidence that an enhancer is physically interacting with a TSSs, which would requires specialized chromatin confirmation capture assays such as HiC, 4C, 5C, etc. However, previous studies have shown that TSSs and enhancers that are close to each other and have highly correlated expression are more likely to be interacting. We can therefore use distance and correlation of expression between TSSs and enhancers to identify TSSs-enhancer links as candidates for physical interactions ^13^.

To do this with CAGEfightR, we first need to indicate the two types of clusters as a factor with two levels:


rowData(RSE)$clusterType <- RSE %>%
    rowData() %>%                  
    use_series("clusterType") %>%  
    as_factor() %>%                
    fct_relevel("TSS")             


We can then calculate all pairwise correlations between TSSs and enhancer within a distance of 50 bp. Here we use the non-parametric Kendall’s tau as a measure of correlation, but other functions for calculating correlation can be supplied (e.g. one could calculate Pearson’s r on log-transformed TPM values to only capture linear relationships):


all_links <- RSE %>%                                                        
    swapRanges %>%                                                          
    findLinks(maxDist = 5e4L,                                               
              directional="clusterType",                                    
              inputAssay="TPM",                                             
              method="kendall")                                             
#> Finding directional links from TSS to Enhancer...                        
#> Calculating 41311 pairwise correlations...                               
#> Preparing output...                                                      
#> # Link summary:                                                          
#> Number of links: 41311                                                   
#> Summary of pairwise distance:                                            
#>    Min. 1st Qu.  Median    Mean 3rd Qu.    Max.                          
#>     205    8832   21307   22341   35060   50000                          
all_links                                                                   
#> GInteractions object with 41311 interactions and 4 metadata columns:     
#>              seqnames1   ranges1        seqnames2   ranges2 | orientation
#>                  <Rle> <IRanges>            <Rle> <IRanges> | <character>
#>       [1]         chr1   6204746 ---         chr1   6226837 |  downstream
#>       [2]         chr1   7079251 ---         chr1   7083527 |  downstream
#>       [3]         chr1   9535519 ---         chr1   9554735 |  downstream
#>       [4]         chr1   9538162 ---         chr1   9554735 |  downstream
#>       [5]         chr1  20941781 ---         chr1  20990601 |  downstream
#>       ...          ...       ... ...          ...       ... .         ...
#>   [41307]  chr9_random    193165 ---  chr9_random    217926 |    upstream
#>   [41308]  chr9_random    193165 ---  chr9_random    242951 |    upstream
#>   [41309]  chr9_random    223641 ---  chr9_random    217926 |  downstream
#>   [41310]  chr9_random    223641 ---  chr9_random    242951 |    upstream
#>   [41311] chrUn_random   3714359 --- chrUn_random   3718258 |    upstream
#>            distance            estimate             p.value              
#>           <integer>           <numeric>           <numeric>              
#>       [1]     22090 -0.0603022689155527   0.805433562909099              
#>       [2]      4275   0.365994211051474   0.128612838399956              
#>       [3]     19215   -0.21320071635561   0.392330339776564              
#>       [4]     16572   0.341121146168977    0.17111237306132              
#>       [5]     48819    0.14070529413629   0.565460671338501              
#>       ...       ...                 ...                 ...              
#>   [41307]     24760   0.477084298221423  0.0423302291213607              
#>   [41308]     49785   0.180906806746658   0.459929012970529              
#>   [41309]      5714 -0.0366987921708787   0.875896057922941              
#>   [41310]     19309  -0.261309831967395    0.28579482541369              
#>   [41311]      3898  -0.170560573084488   0.493773664508106              
#>   -------                                                                
#>   regions: 38454 ranges and 8 metadata columns                           
#>   seqinfo: 35 sequences (1 circular) from mm9 genome                     


The output is a GInteractions-object from the *InteractionSet* package ^23^: For each TSS-enhancer both the distance and orientation (upstream/downstream relative to TSS) is calculated in addition to the correlation estimate and p-value. For now, we are only interested in positive correlations, so we subset and sort the links:


# Subset to only positive correlation       
cor_links <- subset(all_links, estimate > 0)



# Sort based on correlation                                         
cor_links <- cor_links[order(cor_links$estimate, decreasing = TRUE)]


We can then visualize the correlation patterns across a genomic region, here using the most correlated TSS- enhancer link:


# Make plotting region                                      
plot_region <- cor_links[1] %>%                             
    anchors %>%                                             
    GRangesList() %>%                                       
    unlist() %>%                                            
    reduce(ignore.strand=TRUE,                              
           min.gapwidth=1e5) %>%                            
    add(1000)                                               

# Cluster track                                             
cluster_track <- RSE %>%                                    
    subsetByOverlaps(plot_region) %>%                       
    trackClusters(name = "Clusters",                        
                  col = NA,                                 
                  showId=TRUE)                              
#> Setting thick and thin features...                       
#> Merging and sorting...                                   
#> Preparing track...                                       

# Cluster track                                             
link_track <- cor_links %>%                                 
    subsetByOverlaps(plot_region) %>%                       
    trackLinks(name="Links",                                
               interaction.measure = "p.value",             
               interaction.dimension.transform = "log",     
               col.outside="grey",                          
               plot.anchors=FALSE,                          
               col.interactions="black")                    

# Plot at tracks together                                   
plotTracks(list(axis_track,                                 
                link_track,                                 
                cluster_track,                              
                tx_track),                                  
           from = start(plot_region),                       
           to=end(plot_region),                             
           chromosome = as.character(seqnames(plot_region)))


**Figure 7.**
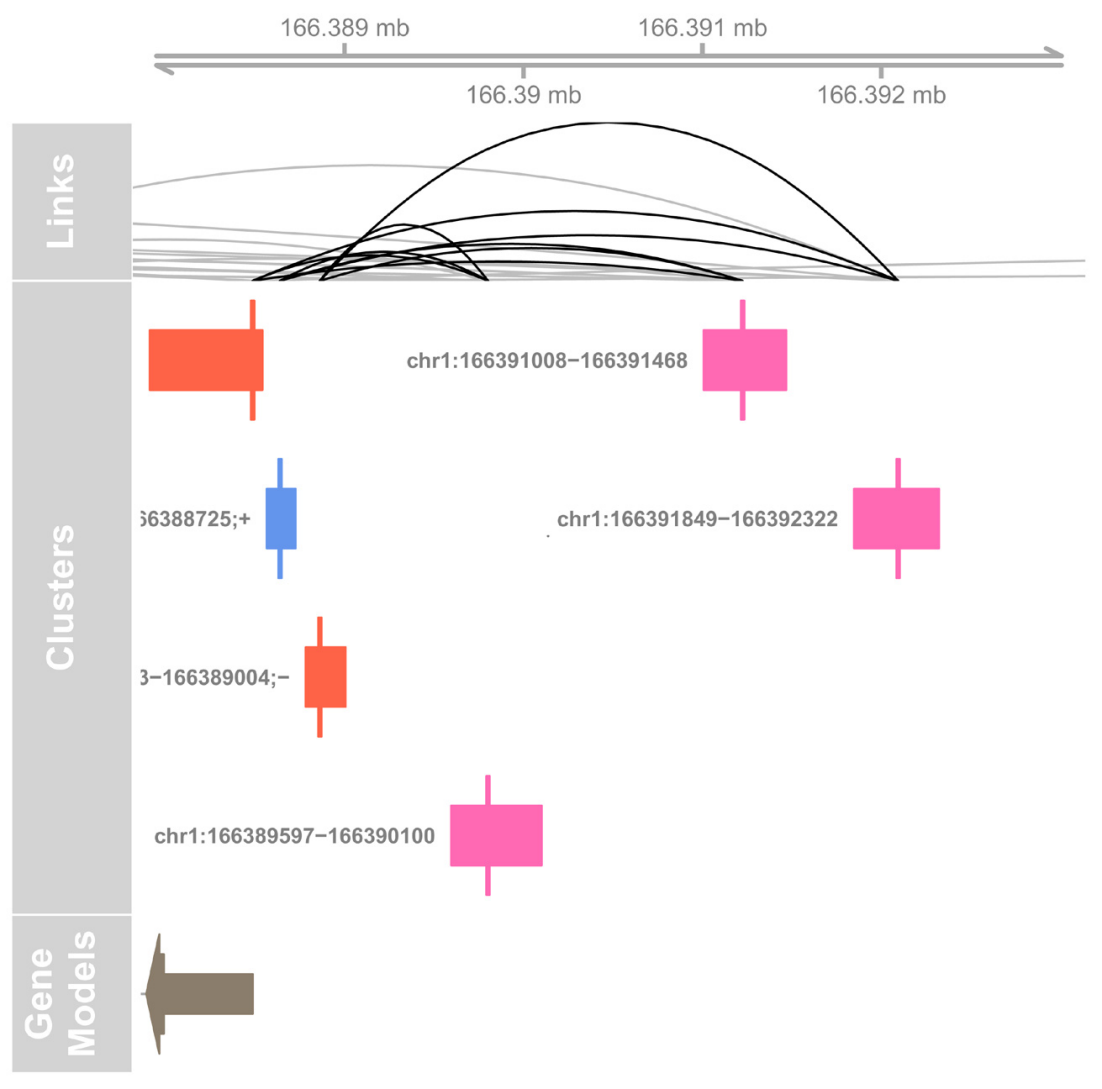
Genome browser example of TSS-enhancer link candidates.

The top track shows the strength of correlations between 3 TSSs around the Atp1b1 gene. The highest correlation is seen between the upstream TSS and the most distal enhancer.

***Finding stretches of enhancers. ***Several studies have found that groups or stretches of closely spaced enhancers tend to show different chromatin characteristics and functions compared to singleton enhancers. Such groups of are often referred to as “super enhancers” or “stretch enhancers” ^34^.

CAGEfightR can detect such *enhancer stretches* based on CAGE data. CAGEfightR groups nearby enhancers into groups and calculates the average pairwise correlation between them, shown below (again using Kendall’s tau):


# Subset to only enhancers
Enhancers <-subset(RSE, clusterType=="Enhancer")

# Find stretches
stretches <-findStretches(Enhancers,
inputAssay ="TPM",
                              
                        mergeDist =12500L,
minSize =5,
                              
                        method ="kendall")
#> Finding stretches...
#> Calculating correlations...
#> # Stretch summary:
#> Number of stretches: 95
#> Total number of clusters inside stretches: 587 / 9943
#> Minimum clusters: 5
#> Maximum clusters: 15
#> Minimum width: 7147
#> Maximum width: 92531
#> Summary of average pairwise correlations:
#>     Min.  1st Qu.   Median     Mean  3rd  Qu.      Max.
#> -0.10038 0.01351 0.08107 0.09097 0.16171 0.37105


Similarly to TSSs and enhancers, we can also annotate stretches based on their relation with known transcripts:


# Annotate                                                             
stretches <- assignTxType(stretches, txModels=txdb)                    
#> Finding hierachical overlaps...                                     
#> ### Overlap summary: ###                                            
#>       txType count percentage                                       
#> 1   promoter    50       52.6                                       
#> 2   proximal     0        0.0                                       
#> 3    fiveUTR     6        6.3                                       
#> 4   threeUTR     5        5.3                                       
#> 5        CDS     3        3.2                                       
#> 6       exon     2        2.1                                       
#> 7     intron    15       15.8                                       
#> 8  antisense     0        0.0                                       
#> 9 intergenic    14       14.7                                       

# Sort by correlation                                                  
stretches <- stretches[order(stretches$aveCor, decreasing=TRUE)]       

# Inspect                                                              
stretches                                                              
#> GRanges object with 95 ranges   and  4 metadata columns:            
#>                               seqnames              ranges  strand |
#>                                  <Rle>           <IRanges>   <Rle> |
#>     chr11:98628005-98647506      chr11   98628005-98647506       * |
#>    chr7:139979437-140003112       chr7 139979437-140003112       * |
#>     chr15:31261340-31279984      chr15   31261340-31279984       * |
#>   chr11:117733009-117752208      chr11 117733009-117752208       * |
#>      chr7:97167988-97188451       chr7   97167988-97188451       * |
#>                         ...        ...                 ...     ... .
#>   chr15:101076561-101093429      chr15 101076561-101093429       * |
#>     chr16:91373912-91399202      chr16   91373912-91399202       * |
#>    chr7:132619265-132644381       chr7 132619265-132644381       * |
#>     chr15:79181690-79208915      chr15   79181690-79208915       * |
#>     chr10:94708643-94729408      chr10   94708643-94729408       * |
#>                                           revmap nClusters          
#>                                    <IntegerList> <integer>          
#>     chr11:98628005-98647506   6600,6601,6602,...         6          
#>    chr7:139979437-140003112   4220,4221,4222,...         5          
#>     chr15:31261340-31279984   7962,7963,7964,...         5          
#>   chr11:117733009-117752208   6785,6786,6787,...         6          
#>      chr7:97167988-97188451   4022,4023,4024,...         6          
#>                         ...                  ...       ...          
#>   chr15:101076561-101093429   8320,8321,8322,...         5          
#>     chr16:91373912-91399202   8643,8644,8645,...         7          
#>    chr7:132619265-132644381   4160,4161,4162,...         5          
#>     chr15:79181690-79208915   8144,8145,8146,...         5          
#>     chr10:94708643-94729408   5823,5824,5825,...         5          
#>                                          aveCor     txType          
#>                                       <numeric>   <factor>          
#>     chr11:98628005-98647506   0.371052840516797   promoter          
#>    chr7:139979437-140003112   0.328630841442886   promoter          
#>     chr15:31261340-31279984   0.301603791540209     intron          
#>   chr11:117733009-117752208   0.284399425439616   promoter          
#>      chr7:97167988-97188451   0.262199740521045   promoter          
#>                         ...                 ...        ...          
#>   chr15:101076561-101093429 -0.0549688493223916 intergenic          
#>     chr16:91373912-91399202 -0.0598361076236999    fiveUTR          
#>    chr7:132619265-132644381 -0.0626248504104628   promoter          
#>     chr15:79181690-79208915 -0.0981772309926707   promoter          
#>     chr10:94708643-94729408  -0.100380656957041     intron          
#>   -------                                                           
#> seqinfo: 35 sequences (1 circular) from mm9 genome                  


The returned GRanges contains the the location, number of enhancers and average correlation for each stretch. Stretches are found in a variety of context, some being intergenic and other spanning various parts of genes. Let us plot one of the top intergenic stretches:


# Make plotting region                                         
plot_region <- stretches["chr17:26666593-26675486"] + 1000     

# Cluster track                                                
cluster_track <- RSE %>%                                       
    subsetByOverlaps(plot_region) %>%                          
    trackClusters(name = "Clusters",                           
                  col = NA,                                    
                  showId=TRUE)                                 
#> Setting thick and thin features...                          
#> Merging and sorting...                                      
#> Preparing track...                                          

# CTSS track                                                   
ctss_track <- CTSSs %>%                                        
    subsetByOverlaps(plot_region) %>%                          
    trackCTSS(name="CTSSs")                                    
#> Splitting pooled signal by strand...                        
#> Preparing track...                                          

# SE track                                                     
stretch_track <- stretches %>%                                 
    subsetByOverlaps(plot_region) %>%                          
    AnnotationTrack(name="Stretches", fill="hotpink", col=NULL)

# Plot at tracks together                                      
plotTracks(list(axis_track,                                    
                stretch_track,                                 
                cluster_track,                                 
                ctss_track),                                   
           from = start(plot_region),                          
           to=end(plot_region),                                
           chromosome = as.character(seqnames(plot_region)))   


**Figure 8.**
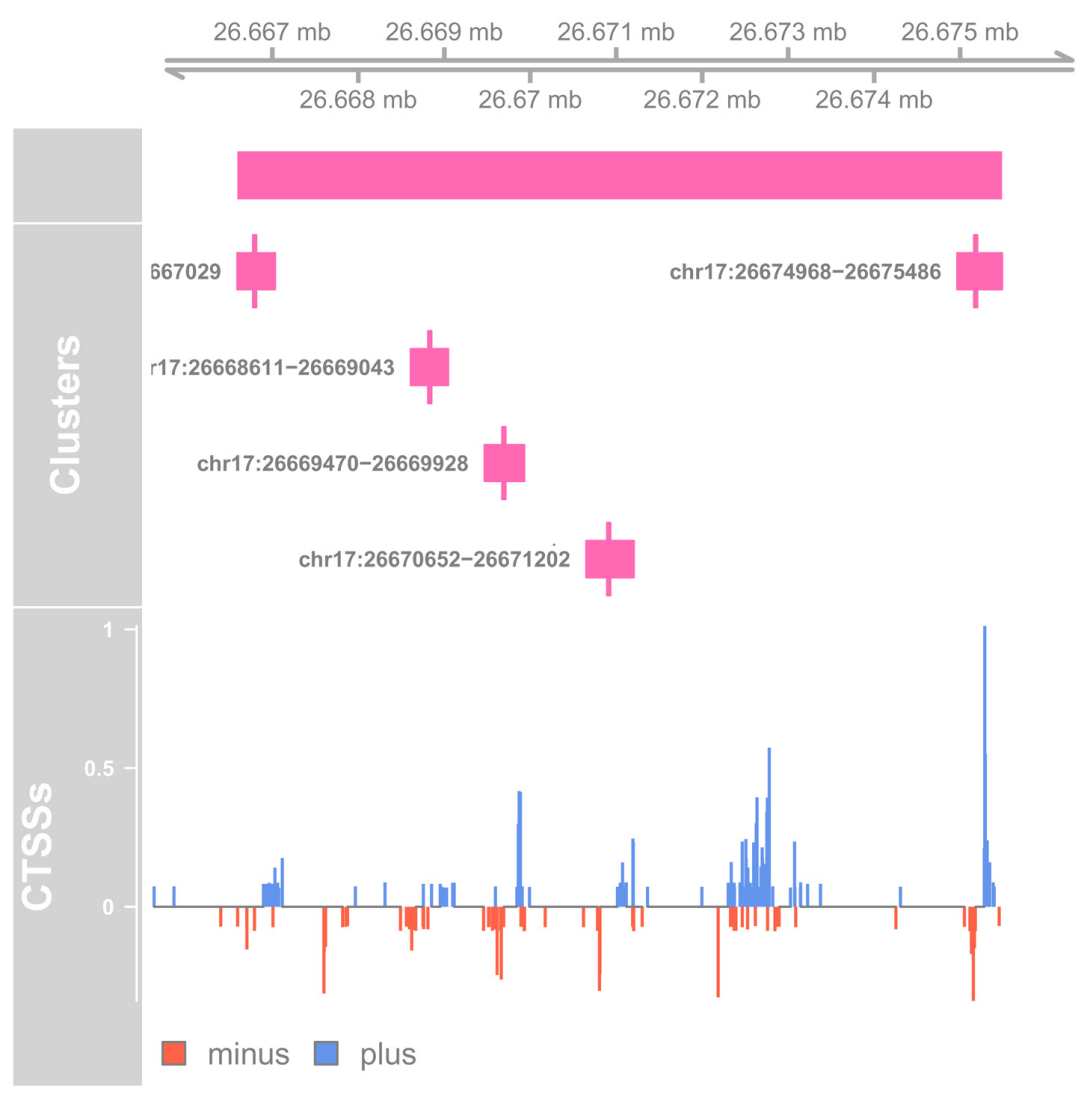
Genome browser example of enhancer stretch.

This stretch is composed of at least 5 enhancers, each of which shows bidirectional transcription.

### Part 3: Differential Expression analysis of TSSs, enhancers and genes

***Normalization of expression and EDA. ***Before performing statistical tests for various measures of Differential Expression (DE), it is important to first conduct a thorough Exploratory Data Analysis (EDA) to identify what factor we need to include in the final model.

Here we will use *DESeq2* ^20^ for normalization and EDA since it offers easy to use functions for performing basic analyses. Other popular tools such as *edgeR* ^21^ and *limma* ^25^ offer similar functionality, as well as more specialized packages for EDA such as *EDASeq*.

DESeq2 offers sophisticated normalization and transformation of count data in the form of the variance stabilized transformation: this adds a dynamic pseudo-count to normalized expression values before log transforming to dampen the inherent mean-variance relationship of count data. This is particularly useful for CAGE data, as CAGE can detect even very lowly expressed TSSs and enhancers.

First, we fit a “blind” version of the variance-stabilizing transformation, since we do not yet know what design is appropriate for this particular study:


# Create DESeq2 object with blank design    
dds_blind <- DESeqDataSet(RSE, design = ~ 1)

# Normalize and log transform               
vst_blind <- vst(dds_blind, blind = TRUE)   


A very useful first representation is a Principal Components Analysis (PCA) plot summarizing variance across the entire experiment:


plotPCA(vst_blind, "Class")


**Figure 9.**
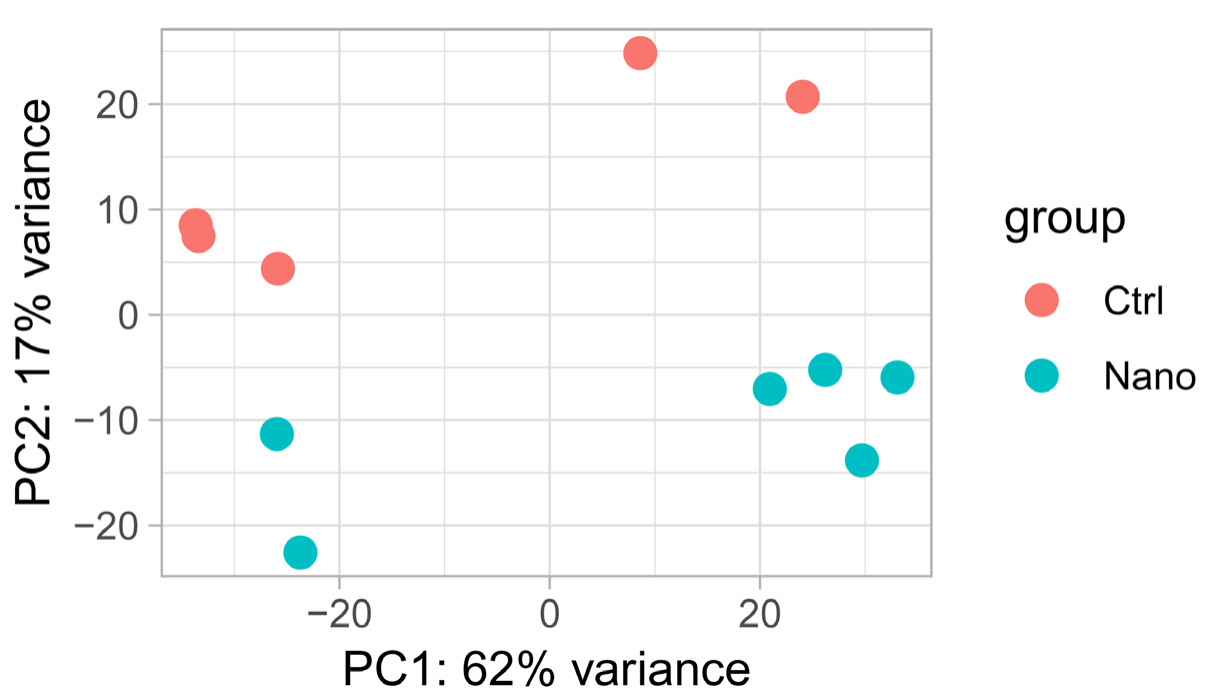
PCA-plot of variance stabilized expression.

We observe that PC2 separates the samples according to the experimental group (control vs nano). However, PC1 also separates samples into two groups. This is suggestive of an unwanted yet systematic effect on expression, often referred as a *batch effect*. We do not want to mistake this unwanted variation for biological variation when we test for differential expression. To prevent this, we can include the batch information as a factor in the final model. Let first define the batch variable:


# Extract pca results                                                     
pca_res <- plotPCA(vst_blind, "Class", returnData=TRUE)                   

# Define a new variable using PC1                                         
batch_var <- ifelse(pca_res$PC1 > 0, "A", "B")                            

# Attach the batch variable as a factor to the experiment                 
RSE$Batch <- factor(batch_var)                                            

# Show the new design                                                     
RSE %>%                                                                   
    colData() %>%                                                         
    subset(select=c(Class, Batch)) %>%                                    
    kable(caption = "Design matrix after adding new batch covariate.") %>%
    kable_styling(latex_options = "hold_position")                        


**Table 4.** Design matrix after adding new batch covariate.

	Class	Batch
C547	Ctrl	B
C548	Ctrl	B
C549	Ctrl	B
C559	Ctrl	A
C560	Ctrl	A
N13	Nano	B
N14	Nano	A
N15	Nano	B
N16	Nano	A
N17	Nano	A
N18	Nano	A

An alternative to manually defining the batch variable, tools such as *sva* and *RUVSeq* can be used to estimate unknown batch effects from the data.

***Cluster-level differential expression. ***Following our short EDA above, we are ready to specify the final design for the experiment: We want to take into account both the Class and Batch of samples:


# Specify design                                  
dds <- DESeqDataSet(RSE, design = ^~^ Batch + Class)

# Fit DESeq2 model                                
dds <- DESeq(dds)                                 
#> estimating size factors                        
#> estimating dispersions                         
#> gene-wise dispersion estimates                 
#> mean-dispersion relationship                   
#> final dispersion estimates                     
#> fitting model and testing                      


We can now extract estimated effects (log fold changes) and statistical significance (p-values) for the Nanovs-Ctrl comparison, implicitly correcting for the batch effect:


# Extract results                                                                  
res <- results(dds,                                                                
               contrast=c("Class", "Nano", "Ctrl"),                                
               alpha=0.05,                                                         
               independentFiltering=TRUE,                                          
               tidy = TRUE) %>%                                                    
    bind_cols(as.data.frame(rowData(RSE))) %>%                                     
    as_tibble                                                                      

# Show the top hits                                                                
res %>%                                                                            
    top_n(-10, padj) %>%                                                           
    dplyr::select(cluster=row,                                                     
                  clusterType,                                                     
                  txType,                                                          
                  baseMean,                                                        
                  log2FoldChange,                                                  
                  padj) %>%                                                        
    kable(caption = "Top differentially expressed TSS and enhancer candidates") %>%
    kable_styling(latex_options = "hold_position")                                 


**Table 5.** Top differentially expressed TSS and enhancer candidates.

cluster	clusterType	txType	baseMean	log2FoldChange	padj
chr1:73977049-73977548;-	TSS	intron	1183.3740	2.838367	0
chr2:32243097-32243468;-	TSS	promoter	30799.5953	3.741789	0
chr3:144423689-144423778;-	TSS	promoter	191.0431	3.709530	0
chr4:125840648-125840820;-	TSS	proximal	1063.4328	3.867574	0
chr4:137325466-137325712;-	TSS	intron	176.7636	3.912592	0
chr7:53971039-53971170;-	TSS	promoter	8720.5204	6.696838	0
chr9:120212846-120213294;+	TSS	promoter	316.0582	2.404706	0
chr11:83222553-83222887;+	TSS	proximal	228.5560	6.098838	0
chr12:105649334-105649472;+	TSS	CDS	175.1364	3.345412	0
chr19:56668148-56668332;+	TSS	CDS	103.8795	-2.254371	0

It always a good idea to inspect a few diagnostics plot to make sure the DESeq2 analysis was successful. One such example is an MA-plot (another useful plot is p-value histogram):


ggplot(res, aes(x=log2(baseMean), y=log2FoldChange, color=padj < 0.05)) +
    geom_point(alpha=0.25) +                                             
    geom_hline(yintercept = 0, linetype="dashed", alpha=0.75) +          
    facet_grid(clusterType^~^.)                                            


**Figure 10.**
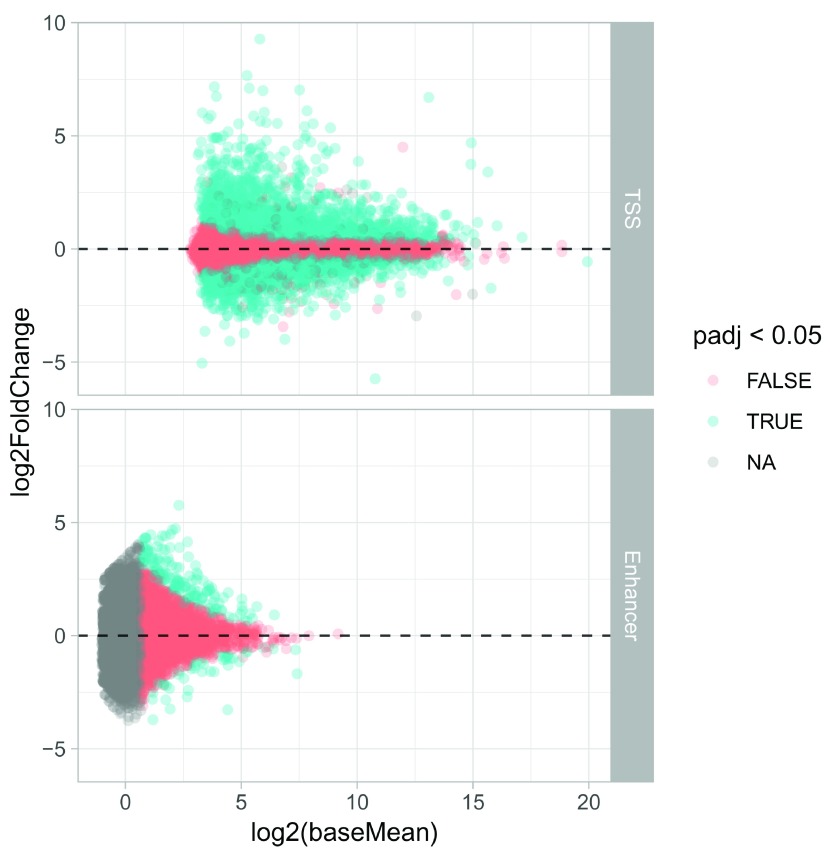
Diagnostic MA plot of the differential expression analysis.

We can see that we overall find more differentially expressed TSSs compared to enhancers, which is expected since they are also more highly expressed. Many enhancers are filtered away for the final DESeq2 analysis (The “Independent Filtering” Step), as their expression level is too low to detect any DE: This increases power for detecting DE at higher expression levels.

We can tabulate the total number of DE TSSs and enhancers:


table(clusterType=rowRanges(RSE)$clusterType, DE=res$padj<0.05)
#>            DE                                               
#> clusterType FALSE  TRUE                                     
#>    TSS      22071  6385                                     
#>    Enhancer  3034   199                                     


***Correcting expression estimates for batch effects. ***In addition to looking at estimates and significance for each cluster, we might also want to look at individual expression values for some top hits. However, we then need to also correct the expression estimates themselves for batch effects, just like we did for log fold changes and p-values (using the same model of course).

Here we use ComBat ^35^ from the *sva* package which is suitable for removing simple batch effects from small experiments. For more advanced setups, removeBatchEffect from limma can remove arbitrarily complex batch effects. The *RUVSeq* package and fsva from sva can be used to remove unknown batch effects.

We again use the variance-stabilizing transformation to prepare the data for ComBat (this makes count data resemble expression estimates obtained from microarrays, as ComBat was originally developed for microarrays).


# Guided variance stabilizing transformation                     
vst_guided <- varianceStabilizingTransformation(dds, blind=FALSE)


To run ComBat we need two additional pieces of information: i) A design matrix describing the biological or wanted effects and ii) the known but unwanted batch effect. We first specify the design matrix, and then run ComBat:


# Design matrix of wanted effects                     
bio_effects <- model.matrix(^~^Class, data=colData(RSE))

# Run ComBat =                                        
assay(RSE, "ComBat") <- ComBat(dat=assay(vst_guided), 
                                    batch=RSE$Batch, # Unwanted batch                     
                                    mod=bio_effects)                                      

#> Found2batches                                      
#> Adjusting for1covariate(s) or covariate level(s)   
#> Standardizing Data across genes                    
#> Fitting L/S model and finding priors               
#> Finding parametric adjustments                     
#> Adjusting the Data                                 


Let us redo the PCA-plot, to see the global effect of the batch effect correction:


# Overwrite assay                        
assay(vst_guided) <- assay(RSE, "ComBat")

# Plot as before                         
plotPCA(vst_guided, "Class")             


**Figure 11.**
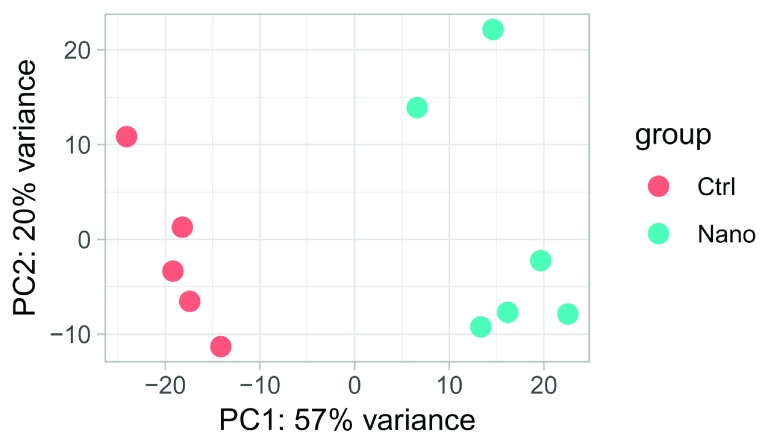
PCA-plot of batch corrected expression.

Now Nano and Ctrl are separated along the first principal component (compared to the second principle component before correction).

Then we extract the top 10 DE enhancers using the following tidyverse code:


# Find top 10 DE enhancers                            
top10 <- res %>%                                      
    filter(clusterType == "Enhancer", padj < 0.05) %>%
    group_by(log2FoldChange >= 0) %>%                 
    top_n(5, wt=abs(log2FoldChange)) %>%              
    pull(row)                                         

# Extract expression values in tidy format            
tidyEnhancers <- assay(RSE, "ComBat")[top10,] %>%     
    t %>%                                             
    as.data.frame %>%                                 
    rownames_to_column("Sample") %>%                  
    mutate(Class=RSE$Class) %>%                       
    gather(key="Enhancer",                            
           value="Expression",                        
           -Sample, -Class,factor_key=TRUE)                           


Finally, we can plot the batch-corrected expression profiles of each individual enhancer:


ggplot(tidyEnhancers, aes(x=Class, y=Expression, fill=Class)) +         
    geom_dotplot(stackdir="center", binaxis="y", dotsize=3) +           
    facet_wrap(~Enhancer, ncol=2, scales="free_y")                      
#> ‘stat_bindot()‘ using ‘bins = 30‘. Pick better value with ‘binwidth‘.


**Figure 12.**
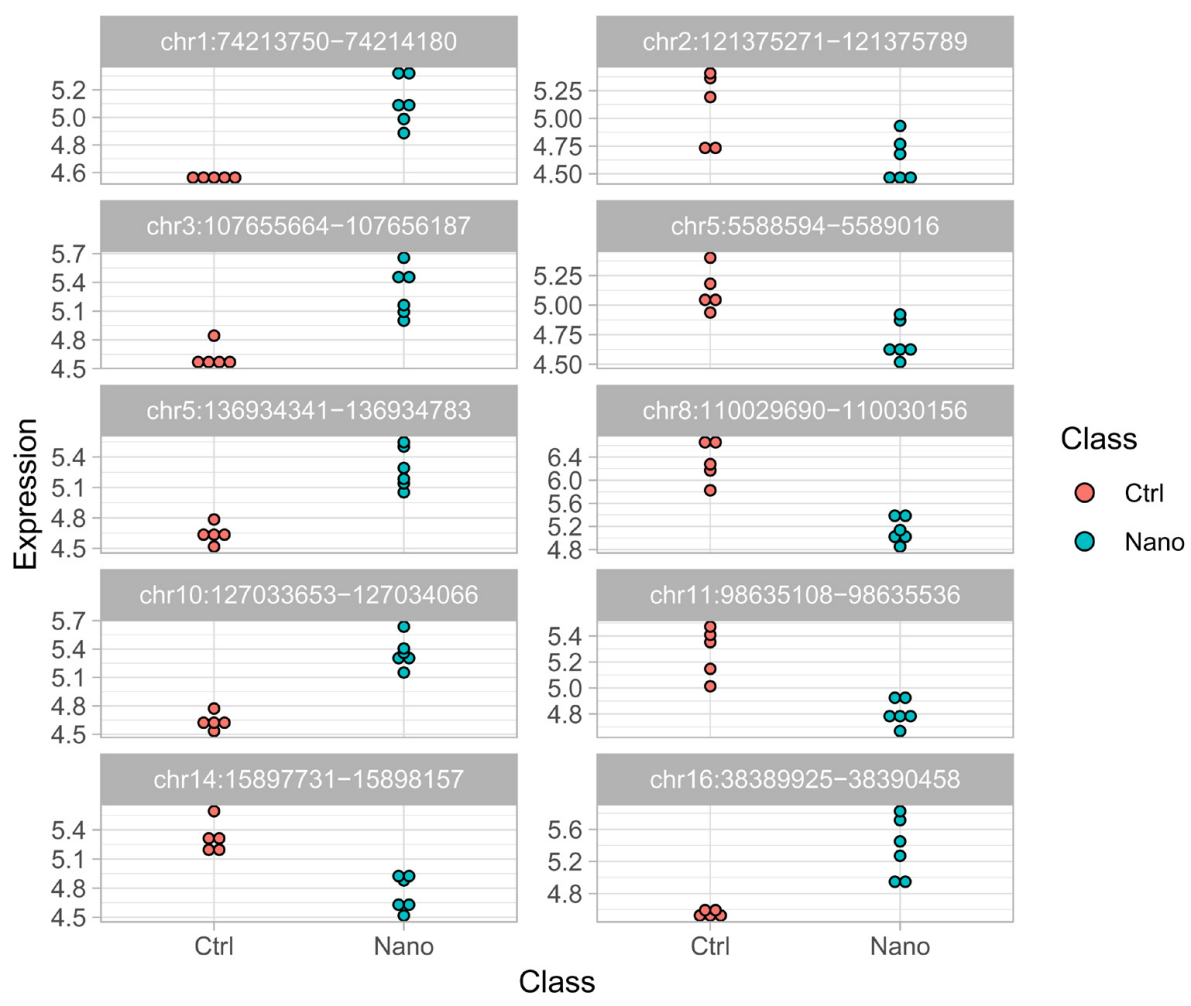
Expression profile of top 10 differentially expressed enhancer candidates.

***Enrichment of DNA-binding motifs. ***A typical question following identification of differentially expressed TSSs and enhancers, is what TFs might be involved in their regulation. To shed light on this question we can annotate TSSs and enhancers with DNA-binding motifs from the JASPAR database ^27^.

First we extract the sequences around TSSs and enhancers. Here we simply define it as +/- 500 bp around TSS peak or enhancer midpoint:


cluster_seqs <- RSE %>%
    rowRanges %>%      
    swapRanges() %>%   
    unstrand() %>%     
    add(500) %>%       
    getSeq(bsg, .)     


Secondly, we use the *TFBSTools* ^36^ package to obtain motifs as Position Frequency Matrices (PFMs) from the *JASPAR2016* database:


# Extract motifs as Position                                        
motif_pfms <- getMatrixSet(JASPAR2016, opts = list(species="10090"))

# Look at the IDs and names of the first few motifs:                
head(name(motif_pfms))                                              
#> MA0004.1 MA0006.1 MA0029.1 MA0063.1 MA0067.1 MA0078.1            
#> "Arnt" "Ahr::Arnt" "Mecom" "Nkx2-5" "Pax2" "Sox17"               


Thirdly, we use the *motifmatchr* package ^37^ to find hits in the sequences:


# Find matches                                             
motif_hits <- matchMotifs(motif_pfms, subject=cluster_seqs)

# Matches are returned as a sparse matrix:                 
motifMatches(motif_hits)[1:5, 1:5]                         
#> 5 x 5 sparse Matrix of class "lgCMatrix"                
#>      MA0004.1 MA0006.1 MA0029.1 MA0063.1 MA0067.1       
#> [1,]        .        .        .        .        |       
#> [2,]        .        .        .        .        .       
#> [3,]        |        .        |        .        .       
#> [4,]        .        .        .        .        .       
#> [5,]        .        |        .        |        .       


Finally we can do a simple Fisher’s Exact test to see if a motif co-occurs more with DE TSSs and enhancer than we would expect be chance. Here we will look at the FOS::JUN motif (MA0099.2):


# 2x2 table for fishers                                     
table(FOSJUN = motifMatches(motif_hits)[,"MA0099.2"],       
      DE = res$padj < 0.05) %>%                             
    print() %>%                                             
    fisher.test()                                           
#>        DE                                                
#> FOSJUN  FALSE TRUE                                       
#>   FALSE 22144 5596                                       
#>   TRUE   2961  988                                       
#>                                                          
#>  Fisher’s Exact Test for Count Data                      
#>                                                          
#> data:  .                                                 
#> p-value = 5.839e-12                                      
#> alternative hypothesis: true odds ratio is not equal to 1
#> 95 percent confidence interval:                          
#>  1.220330 1.427821                                       
#> sample estimates:                                        
#> odds ratio                                               
#>   1.320361                                               


A significant odds ratio above 1 indicate that FOS::JUN is a candidate transcription factor (or, more technically correct, a candidate transcription factor dimer) in regulation of the nanotube response. This is not surprising given that FOS::JUN is part of the TNF-alpha inflammatory pathway (see more below).

Of course, this is a just a very quick and simple analysis of motif enrichment. One could easily have used different regions around TSSs and enhancers and/or split the enrichment analysis between TSSs and enhancers. Other Bioconductor packages like *PWMEnrich*, *rGADEM* and *motifcounter* implements more advanced statistical methods for calculating enrichment of known motifs. *rGADEM*, *BCRANK* and *motifRG* can also be used to calculate enrichment of novel motifs, sometimes referred to as *motif discovery*.

***Gene-level differential expression. ***While CAGE data is naturally analyzed at the level of clusters (TSSs and enhancers) it is in many cases interesting to also look at gene-level expression estimates. A prime example of this is looking at enrichment of Gene Ontology (GO) and Kyoto Encyclopedia of Genes and Genomes (KEGG) terms ^28, 29, 30^ which are only defined at gene-level. CAGEfightR includes functions for annotating clusters with gene models and summarizing expression to gene-level.

We can annotate clusters with gene IDs in the same manner as Transcript IDs:


RSE <- assignGeneID(RSE, geneModels=txdb)                          
#> Extracting genes...                                             
#> Overlapping while taking distance to nearest TSS into account...
#> Finding hierachical overlaps...                                 
#> ### Overlap Summary: ###                                        
#> Features overlapping genes: 81.34 %                             
#> Number of unique genes: 13761                                   


And then use CAGEfightR to sum counts of TSSs within genes:


GSE <- RSE %>%                                        
    subset(clusterType == "TSS") %>%                  
    quantifyGenes(genes="geneID", inputAssay="counts")


The result is RangedSummarizedExperiment where the ranges are a GRangesList holding the TSSs that were summed within each gene:


rowRanges(GSE["100038347",])                                                 
#> GRangesList object of length 1:                                           
#> $100038347                                                                
#> GRanges object with 2 ranges and 9 metadata columns:                      
#>                            seqnames            ranges strand |            
#>                               <Rle>         <IRanges>  <Rle> |            
#>   chr7:80884953-80885056;+     chr7 80884953-80885056      + |            
#>   chr7:80885120-80885677;+     chr7 80885120-80885677      + |            
#>                                     score     thick   support         txID
#>                                 <numeric> <IRanges> <integer>  <character>
#>   chr7:80884953-80885056;+   11.058474477  80885000         5   uc009hrf.2
#>   chr7:80885120-80885677;+ 1162.344739622  80885256        11   uc009hrf.2
#>                              txType   balance bidirectionality clusterType
#>                            <factor> <numeric>        <numeric>    <factor>
#>   chr7:80884953-80885056;+ proximal      <NA>             <NA>         TSS
#>   chr7:80885120-80885677;+ promoter      <NA>             <NA>         TSS
#>                                 geneID                                    
#>                            <character>                                    
#>   chr7:80884953-80885056;+   100038347                                    
#>   chr7:80885120-80885677;+   100038347                                    
#>                                                                           
#> -------                                                                   
#> seqinfo: 35 sequences (1 circular) from mm9 genome                        


The gene IDs in this case is Entrez ID (which is widely used by Bioconductor packages). We can translate these systematic IDs into more human-readable symbols using the *org.Mm.eg.db* annotation package:


# Translate symbols                                        
rowData(GSE)$symbol <- mapIds(odb,                         
                              keys=rownames(GSE),          
                              column="SYMBOL",             
                              keytype="ENTREZID")          
#> ’select()’ returned 1:1 mapping between keys and columns


Having obtained a gene-level count matrix we can now perform gene-level DE analysis. Here we use limma-voom, since limma makes it easy to perform a subsequent enrichment analysis. Other tools such as DESeq2 (above) or edgeR (see below) could also have been used.

**Note**: limma is a powerful tool for DE analysis of count-based data. However, since it depends on log transforming counts, it is not always suitable for analyzing datasets where features have very low counts. This is usually not a problem for gene-level analysis, but can be a problem for enhancers, which are generally very lowly expressed.

Similarly to the DESeq2 analysis, we first build the necessary object and then normalize the expression values:


# Create DGElist object                          
dge <- DGEList(counts=assay(GSE, "counts"),      
               genes=as.data.frame(rowData(GSE)))

# Calculate normalization factors                
dge <- calcNormFactors(dge)                      


Then we apply the voom-transformation to model the mean-variance trend, for which we also need to specify the design matrix (in this case the design must contain both wanted and unwanted effects!). The same design matrix is then used for fitting the gene-wise models:


# Design matrix                                          
mod <- model.matrix(~ Batch + Class, data = colData(GSE))

# Model mean-variance using voom                         
v <- voom(dge, design=mod)                               

# Fit and shrink DE model                                
fit <- lmFit(v, design=mod)                              
eb <- eBayes(fit, robust=TRUE)                           

# Summarize the results                                  
dt <- decideTests(eb)                                    


We can the both report the overall summary of differential gene expression, and look at the first few top hits:


# Global summary                                                          
dt %>%                                                                    
    summary %>%                                                           
    kable(caption="Global summary of differentially expressed genes.") %>%
    kable_styling(latex_options = "hold_position")                        


**Table 6.** Global summary of differentially expressed genes.

	(Intercept)	BatchB	ClassNano
Down	51	2572	1505
NotSig	463	8278	10373
Up	13053	2717	1689


# Inspect top htis                                                 
topTable(eb, coef="ClassNano") %>%                                 
    dplyr::select(symbol, nClusters, AveExpr, logFC, adj.P.Val) %>%
    kable(caption="Top differentially expressed genes.") %>%       
    kable_styling(latex_options = "hold_position")                 


**Table 7.** Top differentially expressed genes.

	symbol	nClusters	AveExpr	logFC	adj.P.Val
66938	Sh3d21	3	5.871004	3.075745	0.0e+00
245049	Myrip	2	4.371325	2.414055	7.0e-07
12722	Clca3a1	1	3.020528	3.692198	7.0e-07
382864	Colq	3	2.770158	-3.426911	1.1e-06
20716	Serpina3n	5	6.384175	1.872782	3.0e-06
72275	2200002D01Rik	2	7.208031	1.693257	5.5e-06
381813	Prmt8	4	4.553612	1.409006	5.8e-06
170706	Tmem37	2	5.503908	1.679690	5.8e-06
18654	Pgf	1	4.862055	2.337045	5.8e-06
20361	Sema7a	1	7.612236	1.473680	5.9e-06

***Enrichment of GO- and KEGG-terms. ***In addition to looking at individual top genes, we can look at how the differentially expressed genes relate to known databases of gene function to gain insight in what biological processes might be affected in the experiment.

limma makes it easy to perform such an enrichment analysis following a DE analysis. As we have gene indexed by Entrez IDs, we can directly use goana to find enriched GO-terms: goana uses a biased urn-model to estimate enrichment of GO-terms, while taking into account the expression levels of DE genes:


# Find enriched GO-terms                                         
GO <- goana(eb, coef = "ClassNano", species = "Mm", trend = TRUE)

# Show top hits                                                  
topGO(GO, ontology = "BP", number = 10) %>%                      
    kable(caption="Top enriched or depleted GO-terms.") %>%      
    kable_styling(latex_options = "hold_position")               


**Table 8.** Top enriched or depleted GO-terms.

	Term	Ont	N	Up	Down	P.Up	P.Down
GO:0006954	inflammatory response	BP	556	142	51	0	0.9562685
GO:0006952	defense response	BP	1072	224	99	0	0.9878373
GO:0097529	myeloid leukocyte migration	BP	170	61	14	0	0.9359984
GO:0010033	response to organic substance	BP	2074	370	196	0	0.9987104
GO:0006950	response to stress	BP	2755	464	246	0	0.9999946
GO:0006955	immune response	BP	1034	210	96	0	0.9833226
GO:0042221	response to chemical	BP	2762	467	292	0	0.9178712
GO:0050900	leukocyte migration	BP	288	83	23	0	0.9792828
GO:0001816	cytokine production	BP	634	143	45	0	0.9998658
GO:0001817	regulation of cytokine production	BP	570	132	39	0	0.9998856

And similarly for KEGG terms:


# Find enriched KEGG-terms
KEGG <-kegga(eb,coef="ClassNano", 
                        species ="Mm", 
                        trend =TRUE)

# Show top hits
topKEGG(KEGG,number =10) 
                        %>%
knitr::kable(caption="Top enriched of depleted KEGG-terms.")%>%
kable_styling(latex_options ="hold_position")


**Table 9.** Top enriched of depleted KEGG-terms.

	Pathway	N	Up	Down	P.Up	P.Down
path:mmu04060	Cytokine-cytokine receptor interaction	173	56	13	0.0000000	0.9579351
path:mmu04668	TNF signaling pathway	105	31	8	0.0000037	0.9186628
path:mmu00600	Sphingolipid metabolism	41	17	2	0.0000051	0.9583011
path:mmu00980	Metabolism of xenobiotics by cytochrome P450	48	4	17	0.8857194	0.0000137
path:mmu03010	Ribosome	122	32	2	0.0000226	0.9999900
path:mmu04064	NF-kappa B signaling pathway	85	24	5	0.0000704	0.9655534
path:mmu04657	IL-17 signaling pathway	74	22	2	0.0000806	0.9985563
path:mmu00982	Drug metabolism - cytochrome P450	46	5	15	0.7266916	0.0001238
path:mmu04630	JAK-STAT signaling pathway	112	29	7	0.0001453	0.9785951
path:mmu04512	ECM-receptor interaction	69	21	13	0.0001488	0.0577601

Both analyses indicate that genes related to the inflammatory response and defense response are upregulated following nanotube exposure. This supports the hypothesis that nanotube induces a response similar to asbestos.

KEGG-terms represents well defined pathways. We can use the *pathview* package ^38^ to investigate in more detail the genes in a given enriched pathway. For example, we can look at regulation of gene in the TNF- signalling pathway:


# Visualize a KEGG
DE_genes <-Filter(
                        function(x) x!=0, 
                        dt[,"ClassNano"])

# This will save a png file to a temporary directory
pathview(DE_genes,species="mmu", 
                        pathway.id="mmu04668", 
                        kegg.dir = tempdir())

# Show the png file
grid.newpage()
grid.raster(png::readPNG(
                        "mmu04668.pathview.png"))


**Figure 13.**
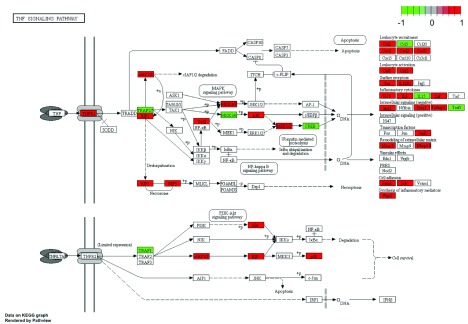
Detailed view of differentially expressed gene in the KEGG TNF-signalling pathway.

***Differential TSS Usage. ***In the above two analyses we looked at whether an individual TSSs or an individual gene was changing expression between experimental groups. However, we might also want to look at whether a gene show differential TSS usage: whether a gene uses different TSSs under different conditions. This problem is similar to differential splicing in RNA-Seq, but looking at TSSs rather than isoforms ^26^. Here we will use the edgeR diffSpliceDGE method to find differential TSS usage, although many other packages could have been used, for example diffSplice from limma, *DEXSeq*, *DRIMSeq*, etc..

Intuitively, diffSpliceDGE tests whether a given TSSs show the same change as other TSSs in the same gene, indicating that TSSs are differentially regulated across the gene. This does however not take into account the relative composition of a given TSSs, e.g. whether a TSS increases from 1%–2% of gene output or 25%–50%. A useful preprocessing step is therefore to filter out TSSs making only a small contribution to total gene expression before analyses.

We use CAGEfightR to remove TSSs that are not expressed as more than 10% of total gene expression in more than 5 samples (We first remove TSSs not assigned to genes):


# Filter away lowly expressed                        
RSE_filtered <- RSE %>%                              
    subset(clusterType == "TSS" & !is.na(geneID)) %>%
    subsetByComposition(inputAssay="counts",         
                        genes="geneID",              
                        unexpressed=0.1,             
                        minSamples=5)                
#> Calculating composition...                        
#> Subsetting...                                     
#> Removed 8001 out of 24500 regions (32.7%)         


We can only do differential TSS usage analysis of genes with multiple TSSs. A useful first visualization is therefore to see how many genes use more than one TSS:


RSE_filtered %>%                                        
    rowData %>%                                         
    as.data.frame %>%                                   
    as_tibble %>%                                       
    dplyr::count(geneID) %>%                            
    ggplot(aes(x = n, fill = n >= 2)) +                 
    geom_bar(alpha=0.75) +                              
    scale_fill_colorblind("Multi-TSS") +                
    labs(x = "Number of TSSs per gene", y = "Frequency")


**Figure 14.**
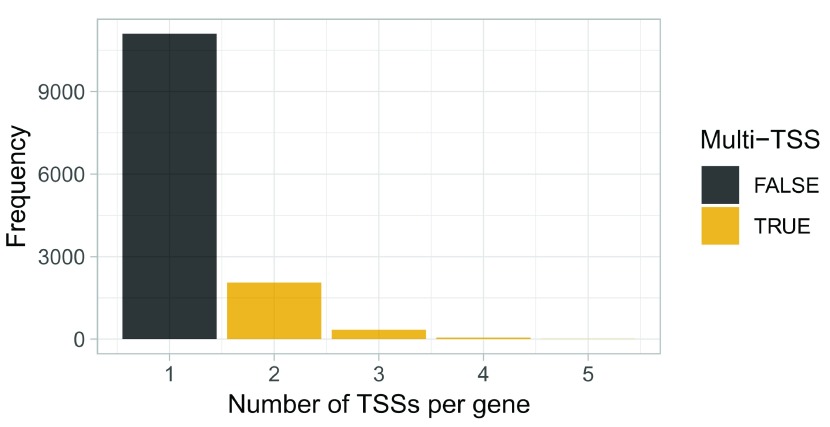
Overview of alternative TSS usage within genes.

While most genes utilize only a single TSSs, many genes use two or more TSSs.

Again, we build the necessary R-objects for running edgeR:


# Annotate with symbols like before:                                     
rowData(RSE_filtered)$symbol <- mapIds(odb,                              
                                       keys=rowData(RSE_filtered)$geneID,
                                       column="SYMBOL",                  
                                       keytype="ENTREZID")               
#> ’select()’ returned 1:1 mapping between keys and columns              

# Extract gene info                                                      
TSS_info <- RSE_filtered %>%                                             
    rowData %>%                                                          
    subset(select=c(score, txType, geneID, symbol)) %>%                  
    as.data.frame                                                        

# Build DGEList                                                          
dge <- DGEList(counts=assay(RSE_filtered, "counts"),                     
               genes=TSS_info)                                           


Then we normalize and fit models using the Quasi-likelihood approach, including the diffSpliceDGE step:


# Estimate normalization factors                                 
dge <- calcNormFactors(dge)                                      

# Estimate dispersion and fit GLMs                               
disp <- estimateDisp(dge, design = mod, tagwise = FALSE)         
QLfit <- glmQLFit(disp, design=mod, robust = TRUE)               

# Apply diffSpliceDGE                                            
ds <- diffSpliceDGE(QLfit, coef = "ClassNano", geneid = "geneID")
#> Total number of exons: 16499                                  
#> Total number of genes: 13563                                  
#> Number of genes with 1 exon: 11098                            
#> Mean number of exons in a gene: 1                             
#> Max number of exons in a gene: 5                              


Now we can look at differential TSS usage at two-levels: Whether an individual TSS shows differential TSS usage (TSS-level) or whether a gene show differential TSS usage in any way (gene-level). First we can look at individual TSSs (TSS-level differential TSS usage):


dtu_TSSs <-topSpliceDGE(ds,test ="exon")
dplyr::select(dtu_TSSs, txType, geneID, symbol, logFC, FDR)%>%
kable(
                        caption ="Top differentially used TSSs")%>%
kable_styling(
                        latex_options ="hold_position")


**Table 10.** Top differentially used TSSs.

	txType	geneID	symbol	logFC	FDR
chr17:13840650-13840851;-	intron	21646	Tcte2	1.7889344	0e+00
chr10:57857044-57857314;+	promoter	110829	Lims1	-1.0651946	0e+00
chr14:70215678-70215876;-	intron	246710	Rhobtb2	2.4933979	0e+00
chr4:141154044-141154185;-	intron	74202	Fblim1	1.7018062	0e+00
chr17:33966135-33966308;+	intron	66416	Ndufa7	2.1612127	0e+00
chr15:76428030-76428201;-	intron	94230	Cpsf1	1.4598815	0e+00
chr19:57271818-57272125;-	promoter	226251	Ablim1	1.1456163	0e+00
chr9:77788968-77789200;+	intron	68801	Elovl5	0.9810692	1e-07
chr11:116395161-116395462;+	proximal	20698	Sphk1	1.7471930	1e-07
chr2:91496305-91496449;+	intron	228359	Arhgap1	0.9809491	3e-07

The interpretation of log fold changes here is slightly different from before: These log fold changes are relative to the overall log fold change for all TSSs in that gene.

Then we can look at results for each gene (Gene-level differential TSS usage):


dtu_genes <-topSpliceDGE(ds,test ="Simes")
dplyr::select(dtu_genes, geneID, symbol, NExons, FDR)%>%
kable(
                        row.names =FALSE,
            
                        caption ="Top genes showing any differential TSS usage.")%>%
kable_styling(latex_options ="hold_position")


**Table 11.** Top genes showing any differential TSS usage.

geneID	symbol	NExons	FDR
21646	Tcte2	4	0e+00
110829	Lims1	3	0e+00
246710	Rhobtb2	3	0e+00
74202	Fblim1	3	0e+00
66416	Ndufa7	3	0e+00
94230	Cpsf1	2	0e+00
226251	Ablim1	3	0e+00
68801	Elovl5	2	1e-07
20698	Sphk1	3	1e-07
228359	Arhgap1	2	2e-07

We see that the two lists agree, which is not surprising given that the gene-level results are obtained by aggregating TSS-level p-values across genes.

We can look at closer at the TSS usage in on of the top hits: We can visualize the batch-corrected expression (See above) of each TSS in the Fblim1 gene via a heatmap:


RSE_filtered %>%                  
    subset(geneID == "74202") %>% 
    assay("ComBat") %>%           
    t %>%                         
    pheatmap(color = magma(100),  
             cluster_cols = FALSE,
             main="Fblim1")       


**Figure 15.**
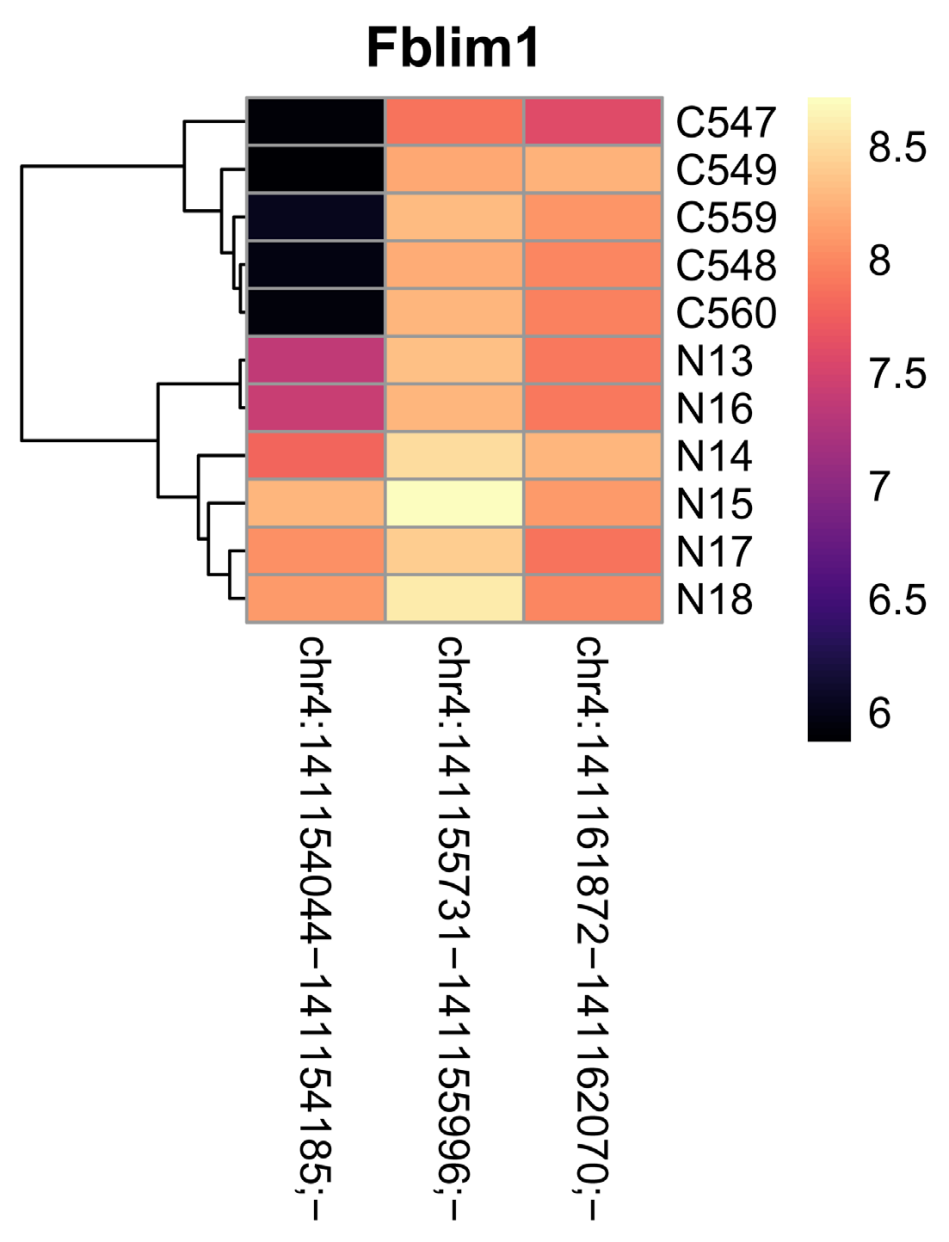
Heatmap showing expression of TSSs within Fblim1.

Fblim1 has 3 TSSs, with 2 of them being used in the Ctrl samples, while the Nano samples also uses the chr4:141154044-141154185;- TSS, as also seen in the TSS-level table above. While a heatmap is useful for seeing expression changes, a genome browser view is better to inspect the genomic context of each TSSs:


# Define plot area                                                     
plot_region <- subset(RSE_filtered, geneID == "74202") %>%             
    rowRanges %>%                                                      
    reduce(min.gapwidth=1e6) %>%                                       
    unstrand() %>%                                                     
    add(5e3L)                                                          

# Create cluster track                                                 
cluster_track <- subsetByOverlaps(RSE_filtered, plot_region) %>%       
    trackClusters(name = "Clusters", col = NA, showId=TRUE)            
#> Setting thick and thin features...                                  
#> Merging and sorting...                                              
#> Preparing track...                                                  

# CTSS tracks for each group                                           
ctrl_track <- subset(CTSSs, select=Class == "Ctrl") %>%                
    calcPooled() %>%                                                   
    subsetByOverlaps(plot_region) %>%                                  
    trackCTSS(name="Ctrl")                                             
#> Warning in calcPooled(.): object already has a column named score in
#> rowData: It will be overwritten!                                    
#> Splitting pooled signal by strand...                                
#> Preparing track...                                                  

nano_track <- subset(CTSSs, select=Class == "Nano") %>%                
    calcPooled() %>%                                                   
    subsetByOverlaps(plot_region) %>%                                  
    trackCTSS(name="Nano")                                             
#> Warning in calcPooled(.): object already has a column named score in
#> rowData: It will be overwritten!                                    
#> Splitting pooled signal by strand...                                
#> Preparing track...                                                  

# Plot at tracks together                                              
plotTracks(list(axis_track,                                            
                tx_track,                                              
                cluster_track,                                         
                Ctrl=ctrl_track,                                       
                nano_track),                                           
           from = start(plot_region),                                  
           to=end(plot_region),                                        
           chromosome = seqnames(plot_region))                         


**Figure 16.**
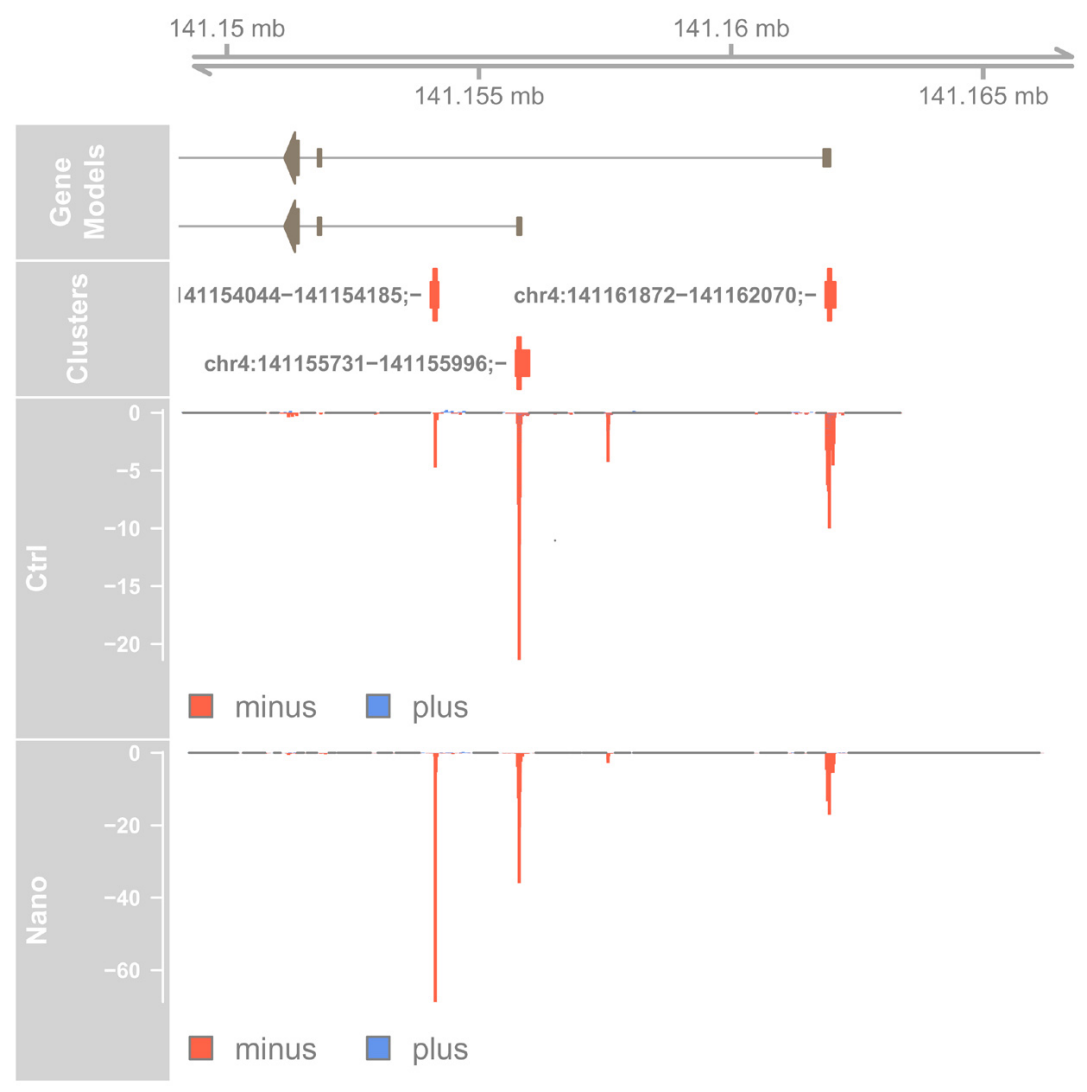
Genome-browser example of differential TSS usage within Fblim1.

The Fblim1 gene uses two annotated TSSs, but the Nano samples also uses a novel intronic TSS.

## Discussion

This workflow is intended as providing an outline of the basic building blocks of CAGE data analysis, going from clustering, to spatial analyses to differential expression. More advanced analyses can be strung together from these basic elements: Finding enhancers linked to DE TSSs, enhancer stretches composed of DE enhancer, comparing DNA binding motif enrichments between DE enhancers and TSSs, etc.

One aspect not covered in this workflow is the utility of CAGE data (and 5’-end data in general) in providing accurate TSSs for studying other types of data. For example, having accurate TSSs is highly beneficial in chromatin research, since the location and nucleosome and TSSs are closely related ^13, 39, 40^. CAGE can be combined with chromatin confirmation assays such as HiC to find new enhancers that are both co-expressed and physically interacting with TSSs. Many genome-wide association studies are finding that disease-related genetic variants are found in intergenic regions, that are often poorly annotated. The accurate enhancer locations provided by CAGE can greatly aid interpretation of such variants ^41^. The adherence of CAGEfightR to standard Bioconductor classes facilitates these inter-assay analyses by making it easy to mix-and-match multiple packages developed for different experimental assays.

## Software and data availability

The following software versions were used in this article:

- **R version**: R version 3.6.0 (2019-04-26)
- **Bioconductor version**: 3.9
- **CAGEfightR version**: 1.4.0

CAGEWorkflow: https://doi.org/10.18129/B9.bioc.CAGEWorkflow ^42^

License: GPL-3

Mouse nanotube CAGE data: https://doi.org/10.18129/B9.bioc.nanotubes ^24^

License: GPL-3

## References

[1] Smale ST Kadonaga JT: The RNA polymerase II core promoter. *Annu Rev Biochem.* 2003;72(1):449–479. 10.1146/annurev.biochem.72.121801.161520 12651739

[2] Kadonaga JT: Perspectives on the RNA polymerase II core promoter. *Wiley Interdiscip Rev Dev Biol.* 2012;1(1):40–51. 10.1002/wdev.21 23801666 PMC3695423

[3] Lenhard B Sandelin A Carninci P: Metazoan promoters: emerging characteristics and insights into transcriptional regulation. *Nat Rev Genet.* 2012;13(4):233–45. 10.1038/nrg3163 22392219

[4] Haberle V Stark A: Eukaryotic core promoters and the functional basis of transcription initiation. *Nat Rev Mol Cell Biol.* 2018;19(10):621–637. 10.1038/s41580-018-0028-8 29946135 PMC6205604

[5] Adiconis X Haber AL Simmons SK: Comprehensive comparative analysis of 5'-end RNA-sequencing methods. *Nat Methods.* 2018;15(7):505–511. 10.1038/s41592-018-0014-2 29867192 PMC6075671

[6] Takahashi H Kato S Murata M: CAGE (cap analysis of gene expression): a protocol for the detection of promoter and transcriptional networks. *Methods Mol Biol.* 2012;786(3C):181–200. 10.1007/978-1-61779-292-2_11 21938627 PMC4094367

[7] Carninci P Sandelin A Lenhard B: Genome-wide analysis of mammalian promoter architecture and evolution. *Nat Genet.* 2006;38(6):626–35. 10.1038/ng1789 16645617

[8] Sandelin A Carninci P Lenhard B: Mammalian RNA polymerase II core promoters: insights from genome-wide studies. *Nat Rev Genet.* 2007;8(6):424–436. 10.1038/nrg2026 17486122

[9] Kawaji H Lizio M Itoh M: Comparison of CAGE and RNA-seq transcriptome profiling using clonally amplified and single-molecule next-generation sequencing. *Genome Res.* 2014;24(4):708–717. 10.1101/gr.156232.113 24676093 PMC3975069

[10] FANTOM Consortium and the RIKEN PMI and CLST (DGT), Forrest AR Kawaji H: A promoter-level mammalian expression atlas. *Nature.* 2014;507(7493):462–70. 10.1038/nature13182 24670764 PMC4529748

[11] Hon CC Ramilowski JA Harshbarger J: An atlas of human long non-coding RNAs with accurate 5' ends. *Nature.* 2017;543(7644):199–204. 10.1038/nature21374 28241135 PMC6857182

[12] Kim TK Hemberg M Gray JM: Widespread transcription at neuronal activity-regulated enhancers. *Nature.* 2010;465(7295):182–7. 10.1038/nature09033 20393465 PMC3020079

[13] Andersson R Gebhard C Miguel-Escalada I: An atlas of active enhancers across human cell types and tissues. *Nature.* 2014;507(7493):455–61. 10.1038/nature12787 24670763 PMC5215096

[14] Huber W Carey VJ Gentleman R: Orchestrating high-throughput genomic analysis with Bioconductor. *Nat Methods.* 2015;12(2):115–121. 10.1038/nmeth.3252 25633503 PMC4509590

[15] Raborn RT Brendel VP Sridharan K: TSRchitect: Promoter identification from large-scale TSS profiling data. 10.18129/B9.bioc.TSRchitect

[16] Bhardwaj V: icetea: Integrating Cap Enrichment with Transcript Expression Analysis, 2019. Reference Source

[17] Haberle V Forrest AR Hayashizaki Y: *CAGEr*: precise TSS data retrieval and high-resolution promoterome mining for integrative analyses. *Nucleic Acids Res.* 2015;43(8):e51. 10.1093/nar/gkv054 25653163 PMC4417143

[18] Thodberg M Thieffry A Vitting-Seerup K: CAGEfightR: Cap Analysis of Gene Expression (CAGE) in R/Bioconductor. *bioRxiv.* 2018; 310623. 10.1101/310623

[19] Frith MC Valen E Krogh A: A code for transcription initiation in mammalian genomes. *Genome Res.* 2008;18(1):1–12. 10.1101/gr.6831208 18032727 PMC2134772

[20] Love MI Huber W Anders S: Moderated estimation of fold change and dispersion for RNA-seq data with DESeq2. *Genome Biol.* 2014;15(12):550. 10.1186/s13059-014-0550-8 25516281 PMC4302049

[21] Robinson MD McCarthy DJ Smyth GK: edgeR: a Bioconductor package for differential expression analysis of digital gene expression data. *Bioinformatics.* 2010;26(1):139–40. 10.1093/bioinformatics/btp616 19910308 PMC2796818

[22] Lawrence M Huber W Pagès H: Software for computing and annotating genomic ranges. *PLoS Comput Biol.* 2013;9(8):e1003118. 10.1371/journal.pcbi.1003118 23950696 PMC3738458

[23] Lun AT Perry M Ing-Simmons E: Infrastructure for genomic interactions: Bioconductor classes for Hi-C, ChIA-PET and related experiments [version 2; peer review: 2 approved]. *F1000Res.* 2016;5:950. 10.12688/f1000research.8759.2 27303634 PMC4890298

[24] Bornholdt J Saber AT Lilje B: Identification of Gene Transcription Start Sites and Enhancers Responding to Pulmonary Carbon Nanotube Exposure *in Vivo*. *ACS Nano.* 2017;11(4):3597–3613. 10.1021/acsnano.6b07533 28345861

[25] Ritchie ME Phipson B Wu D: *limma* powers differential expression analyses for RNA-sequencing and microarray studies. *Nucleic Acids Res.* 2015;43(7):e47. 10.1093/nar/gkv007 25605792 PMC4402510

[26] Soneson C Love MI Robinson MD: Differential analyses for RNA-seq: transcript-level estimates improve gene-level inferences [version 2; peer review: 2 approved]. *F1000Res.* 2015;4:1521. 10.12688/f1000research.7563.2 26925227 PMC4712774

[27] Mathelier A Fornes O Arenillas DJ: JASPAR 2016: a major expansion and update of the open-access database of transcription factor binding profiles. *Nucleic Acids Res.* 2016;44(D1):D110–D115. 10.1093/nar/gkv1176 26531826 PMC4702842

[28] Ashburner M Ball CA Blake JA: Gene ontology: tool for the unification of biology. The Gene Ontology Consortium. *Nat Genet.* 2000;25(1):25–9. 10.1038/75556 10802651 PMC3037419

[29] The Gene Ontology Consortium: The Gene Ontology Resource: 20 years and still GOing strong. *Nucleic Acids Res.* 2019;47(D1):D330–D338. 10.1093/nar/gky1055 30395331 PMC6323945

[30] Kanehisa M Goto S: KEGG: kyoto encyclopedia of genes and genomes. *Nucleic Acids Res.* 2000;28(1):27–30. 10.1093/nar/28.1.27 10592173 PMC102409

[31] Hahne F Ivanek R: Visualizing Genomic Data Using Gviz and Bioconductor. *Methods Mol Biol.*Springer New York, New York, NY.2016;1418:335–351. 10.1007/978-1-4939-3578-9_16 27008022

[32] Schneider TD Stephens RM: Sequence logos: a new way to display consensus sequences. *Nucleic Acids Res.* 1990;18(20):6097–100. 10.1093/nar/18.20.6097 2172928 PMC332411

[33] Wagih O: ggseqlogo: a versatile R package for drawing sequence logos. *Bioinformatics.* 2017;33(22):3645–3647. 10.1093/bioinformatics/btx469 29036507

[34] Pott S Lieb JD: What are super-enhancers? *Nat Genet.* 2015;47(1):8–12. 10.1038/ng.3167 25547603

[35] Johnson WE Li C Rabinovic A: Adjusting batch effects in microarray expression data using empirical Bayes methods. *Biostatistics.* 2007;8(1):118–27. 10.1093/biostatistics/kxj037 16632515

[36] Tan G Lenhard B: TFBSTools: an R/bioconductor package for transcription factor binding site analysis. *Bioinformatics.* 2016;32(10):1555–1556. 10.1093/bioinformatics/btw024 26794315 PMC4866524

[37] Schep A: motifmatchr: Fast Motif Matching in R.2018 10.18129/B9.bioc.motifmatchr

[38] Luo W Brouwer C: Pathview: an R/Bioconductor package for pathway-based data integration and visualization. *Bioinformatics.* 2013;29(14):1830–1831. 10.1093/bioinformatics/btt285 23740750 PMC3702256

[39] Duttke SHC Lacadie SA Ibrahim MM: Human promoters are intrinsically directional. *Mol Cell.* 2015;57(4):674–684. 10.1016/j.molcel.2014.12.029 25639469 PMC4336624

[40] Thodberg M Thieffry A Bornholdt J: Comprehensive profiling of the fission yeast transcription start site activity during stress and media response. *Nucleic Acids Res.* 2019;47(4):1671–1691. 10.1093/nar/gky1227 30566651 PMC6393241

[41] Boyd M Thodberg M Vitezic M: Characterization of the enhancer and promoter landscape of inflammatory bowel disease from human colon biopsies. *Nat Commun.* 2018;9(1):1661. 10.1038/s41467-018-03766-z 29695774 PMC5916929

[42] Thodberg M: CAGEWorkflow: A step-by-step guide to analyzing CAGE data using R/Bioconductor. R package version 1.0.0.2019 10.18129/B9.bioc.CAGEWorkflow PMC6613478 31327999

