# Supplementary material for: A step-by-step guide to analyzing CAGE data using R/Bioconductor
Source: F1000Res. 2019 Jun 18;8:886. [Version 1] doi: 10.12688/f1000research.18456.1 (PMC6613478; doi:10.12688/f1000research.18456.1)
Supplement: Peer review history

# Peer review: Reviewer response for version 1

**DOI:** 10.5256/f1000research.20193.r50326

**Published:** 2019-07-10

**Authors:** Nevena Cvetesic

**Affiliations:** 1 Institute of Clinical Sciences (ICS), Faculty of Medicine, Imperial College London, London, UK; 2 MRC London Institute of Medical Sciences, London, UK

Thodberg and Sandelin present CAGEfightR, a new Bioconductor package suited for analysis of 5'end oriented datasets, derived from CAGE-seq and similar techniques. 

 Although similar Bioconductor packages exist (icetea, CAGEr, TSRchitect), the greatest strength of CAGEfightR is unique in its ability to call or predict putative enhancers based on the bidirectional transcription initiation signature, thus filling the gap in the current R-based toolkit for CAGE-like data analysis.

 In addition, using CAGEfightR, hypotheses of enhancer-promoter interactions based on co-expression levels can be easily set and visualised.

 The package is well-documented, and the step-by-step protocol well written and easy to follow. I only have a few minor comments which could benefit the general reader:

1. The introduction and abstract could state more clearly that CAGE allows TSS mapping of only RNA polymerase II transcripts. Though this is implied through usage of cap-trapping, I would keep in mind that this workflow might be used by general readers not so familiar with TSS-mapping techniques. Considering the advent of technologies that capture RNA polI-RNApolIII transcripts, expected to be much noisier, it would be better to make it as clear as possible. If the authors believe CAGEfightR could be of use on noisier data, from experimental methods based on negative selection (such as TSS-seq), it would be worth testing this and including a few sentences, as this would promote CAGEfightR usage on any TSS mapping technique.
2. I support comparison of existing packages in the form of Table 1; however, I would expand this comparison to include unique features that perhaps CAGEfightR does not have - e.g. icetea and TSRchitect support paired-end data, CAGEr has TSS-shifting discovery function and implemented G-correction function to remove mismatching G’s from 5’ends of reads.
3. Common problem with CAGE and CAGE-like data which is obtained through reverse transcription, is the addition of a G, or so called G-bias upstream of the true transcription start site.
  It would be beneficial for general readers as this is a step-by-step protocol to discuss how to generate BigWig files from fastq files, and how to address the G-bias problem/i.e. remove the mismatching G’s at the 5’end of the reads.
4. The authors prefer to use the term TSS regions or TSSs in place of tag clusters as aggregated CTSSs, and even TSSs as a broader term for CTSS while CTSS is just a CAGE-supported TSS. This becomes very confusing for a general reader, especially page 36:
  “Now we look at differential TSS usage at two-levels: Whether an individual TSS shows differential TSS usage (TSS-level) or whether a gene show differential TSS usage in any way (gene-level). First we can look at individual TSSs (TSS-level differential TSS usage).”
  I would suggest usage of CTSS for individual CAGE-supported TSSs, tag cluster - for an aggregate of individual CTSSs based on distance based clustering or whatever methodology, and tag cluster can be interchangeably used with promoter where needed.
5. Interquartile range should probably be interquantile range as it spans middle 10-90th percentile of the signal. It would also be beneficial to explain why is it used instead of all signal (more robust measure that excludes outliers and is less sensitive to sequencing depth etc).
6. I am a bit surprised the authors use such harsh filtering step before plotting distributions of IQR (Figure 5, TPM >=10), I would assume that the problem is in tag clusters which seem sharp - single bp, and therefore it would perhaps be beneficial to add a more stringent filtering step only to single bp tag clusters to be retained only if highly expressed (>= 5 TPM), while a lower threshold can be applied on broad tag clusters as multiple CTSS within a tag cluster give more certainty that it is not just noise.

I confirm that I have read this submission and believe that I have an appropriate level of expertise to confirm that it is of an acceptable scientific standard.

# Peer review: Reviewer response for version 1

**DOI:** 10.5256/f1000research.20193.r50076

**Published:** 2019-07-05

**Authors:** Kenta Nakai, Luis Augusto Eijy Nagai

**Affiliations:** 1 The Institute of Medical Science, The University of Tokyo, Tokyo, Japan

In this paper, the authors present a cookbook for analyzing CAGE data mainly through their own Bioconductor package, CAGEfightR, applied to sample data, which were analyzed and published by their group previously. By following the given source codes, readers will be able to learn how they can obtain various kinds of information rather easily. Thus, I feel that this publication will be useful for those who want to learn how to analyze CAGE data quickly. On the other hand, it will not tell us how to solve our deeper problems in research. I know that this is out of the scope of this tutorial but I can’t help but feeling, for example, how the given procedure for reducing batch effects can be justified (see below). Thus, here are some of my suggestions for its further improvement:

1. In our realistic situations, the EDA approach is quite important. In this sense, I appreciate their demonstration on how to remove batch effects from the expression data between various samples, using ComBat (Figs. 9 and 11). However, most researchers will not be satisfied with just seeing that the PC1 has become to separate positive and negative groups; it is natural that they would like to confirm if the correction was enough or not; they would also wish to see what the new PC2 as well as the old PC1 represent. Therefore, I recommend the authors to extend Table 2 for characterizing each sample from various features (e.g., experimental conditions and data size) and to use such features for the interpretation. More discussion and/or additional attempts to clarify the most probable main reason for the initial batch effects would be desirable.
2. Similarly, since the first author does not seem to have been a member of the previous analysis, it is interesting to see the consistency between the two studies. For example, the observation that inflammation-related genes were activated seems to be the same in both analyses. Then, are the genes with differential TSSs likely to explain the phenomenon? How much are the detected enhancers contributing to the differential expression? Do these enhancers (or newly activated TSSs) share any over-represented motifs? From the same reason, I recommend the authors to avoid using (ugly) chromosomal coordinates to represent genes/promoters/enhancers, wherever possible. It would be great if the authors can show that they could perform deeper analyses this time.
3. For the convenience of wider readers, it might be useful to show the way how to obtain BigWig files from rawer data (BAM or fastq, if possible).
4. Similarly, a summary table of used tools (except CAGEfightR), containing their input file information as well as their main purposes might be useful.
5. Also, it might be useful if there is a summary on what CAGEfightR can do/cannot do. For example, is it possible to combine different sources of CAGE data with this workflow?
6. One of my students tried to follow the workflow. At first, she failed to install some of the packages. It was because the version of R she used was R3.5. Thus, this point should be clearly noted. In addition, she reports that to run the code “Fit and shrink DE model”, the installation of “statmod” and “BiasedUrn” was necessary. Perhaps, some additional information on how to setup initial environment would be useful for beginners.
7. As a cookbook, it is desirable that users can find their necessary items more easily (via a table of contents with clearer headers, perhaps?).
8. There seems to be some confusion on the versions used:
  As for the mouse genome sequence, (1) both mm9 and mm10 are used. (2) Why didn’t they use the latest version of JASPAR (Jaspar2018)?
9. The manuscript seems to contain many typos. Here are some that we found (there are likely to be more): (1)This is workflow is a case study on (2) CAGE dusters (3) can be speed up (4) novel TSSs are expresse d (5) to try infer (6 )this is a just a very quick (7) The returned GRanges contains the the location (8) going from clustering, to spatial analyses to differential expression.

We confirm that we have read this submission and believe that we have an appropriate level of expertise to confirm that it is of an acceptable scientific standard.

# Peer review: Reviewer response for version 1

**DOI:** 10.5256/f1000research.20193.r50074

**Published:** 2019-06-24

**Authors:** Aaron Lun

**Affiliations:** 1 Genentech, Inc., South San Francisco, CA, USA

Thodberg and Sandelin describe a comprehensive workflow for the basic analysis of CAGE data. The workflow is simple, well-presented and the code mostly runs without problems. I only have a few minor comments to improve its usefulness for the general reader:

- There should be a general overview on how the CTSS BigWig files are generated from raw FASTQ files.
- Consider using ExperimentHub for the nanotubes package. This provides greater control over which files are downloaded, rather than forcing the user to obtain all files at installation. This is especially useful if you have multiple data sets - see, for example, the chipseqDBData package. ^1^
- Explain what a BigWigFileList is, and why it needs to be used instead of having a simple character vector.
- Consider not having string'ified intervals as the row names in the output of quickTSSs(). In some situations, generation of the strings can use more RAM than the actual data itself. It definitely slows down any attempt to 'show' the output. I would suggest that this be deferred as late as possible, e.g., until someone needs the strings as row names of a data frame to save to file.
- More details are required on how quantification is performed. For example, I assume counts are summed directly from single strands for TSSs. For enhancers, are counts summed from both strands? 
- "As enhancers are the most complicated type, we keep only enhancers if a TSS and enhancer overlaps:" The complexity of the enhancers doesn't really provide a motivation for only keeping enhancers in cases of overlaps. The better reason is that all enhancers would be detected as two TSSs if the strandedness was ignored; if they do overlap, it would be more appropriate to interpret them as a single enhancer rather than as two distinct TSS events.
- The Interquartile Range (IQR)... of what? I assume that the range refers to the length of the interval that contains 10 to 90% of a TSS's counts. Incidentally, the IQR is no longer an IQR if it's redefined from 10-90%.
- The single quotes in the highTSSs call are malformed, which prevents copy-pasting.
- One could consider using a 2-component mixture model to classify elements into sharp/broad in a more automated manner.
- I presume that the pairwise correlations for the TSS-enhancer interactions are computed across samples for each TSS/enhancer pair. If so, is this done after blocking on the design? Otherwise it is possible to obtain strong positive correlations simply because a TSS and the enhancer happen to respond in the same direction to a given treatment. If there is a genuine physical interaction, it should manifest as correlations within each treatment condition, where stochastic differences in RNA polymerase activity affect both the TSS and its interacting enhancer.
- There is no correction for multiple testing in the p-values from the links. While I recognise that this is a limitation of the small sample size, it should still be pointed out as a caveat of the analysis.
- All normalization steps in the DE analyses assume that most of the input features are not differentially expressed between conditions. This is usually not a concern, but if aggressive feature selection is performed, it may become a problem. For example, if one were to perform the DE analysis on superenhancers only, it would give incorrect results in situations where the superenhancer activity increases globally upon treatment.

I confirm that I have read this submission and believe that I have an appropriate level of expertise to confirm that it is of an acceptable scientific standard.

## References

[1] : From reads to regions: a Bioconductor workflow to detect differential binding in ChIP-seq data. *F1000Res*.2015;4: 10.12688/f1000research.7016.2 1080 10.12688/f1000research.7016.2 26834993PMC4706055
